# Vinegar Volatile Organic Compounds: Analytical Methods, Constituents, and Formation Processes

**DOI:** 10.3389/fmicb.2022.907883

**Published:** 2022-06-30

**Authors:** Zhenzhen Xie, Chanisara Koysomboon, Huan Zhang, Zhenming Lu, Xiuyan Zhang, Fusheng Chen

**Affiliations:** ^1^Hubei International Scientific and Technological Cooperation Base of Traditional Fermented Foods, Huazhong Agricultural University, Wuhan, China; ^2^College of Food Science and Technology, Huazhong Agricultural University, Wuhan, China; ^3^National Engineering Research Center for Cereal Fermentation and Food Biomanufacturing, Jiangnan University, Wuxi, China

**Keywords:** vinegar, fermentation process, volatile organic compound, analytical method, producing mechanism

## Abstract

Vinegar is an acid condiment shared all over the world. According to the raw materials, vinegar can be mainly divided into fruit and cereal ones, both of which possess unique aroma and flavor characteristics and corresponding volatile organic compounds (VOCs). Many studies about vinegar VOCs' (VVOCs) sorts, analytical methods, and forming mechanisms have been done. In this review, the main categories of vinegar and their distribution in the world are briefly introduced, then VVOCs' analytical and identified methods, types, and forming processes are summarized. Additionally, the VVOCs' research directions are discussed and prospected. According to the searched literatures, this study is the first to systematically review the analytical methods, sorts, and formation mechanisms of VVOCs, which will make the readers better understand the vinegar's aromas and flavors and their producing mechanisms.

Vinegar is one of the few common condiments all over the world. Based on the raw materials, vinegar can be mainly classified into fruit and cereal ones. Cereal vinegar's raw materials are mainly sorghum, rice, wheat, or other materials rich in starch, while fruit vinegar's raw materials mainly include various fruits, such as grape, apple, pineapple, and so on. Almost all the countries or regions in the world have their own kinds of vinegar with unique aromas and flavors, which come from the relative raw materials, microorganisms, and production processes with vinegar (Solieri and Giudici, 2009; Matsushita et al., 2016; Sengun, 2017). Besides seasonings, vinegar is also consumed as functional food and drink since some healthful compounds are found in it, especially in the traditional ones, which are summarized in our previous review (Chen et al., 2016). In this review, we first briefly introduce the vinegar classification and distribution in the world, then the advance in the analytical methods and the formation mechanisms of vinegar volatile organic compounds (VVOCs) are described in detail. In the end, the prospects to investigate VVOCs and their production mechanisms are also discussed and prospected.

## Introduction of Vinegar in the World

### Fruit Vinegar

**Figure 1:**
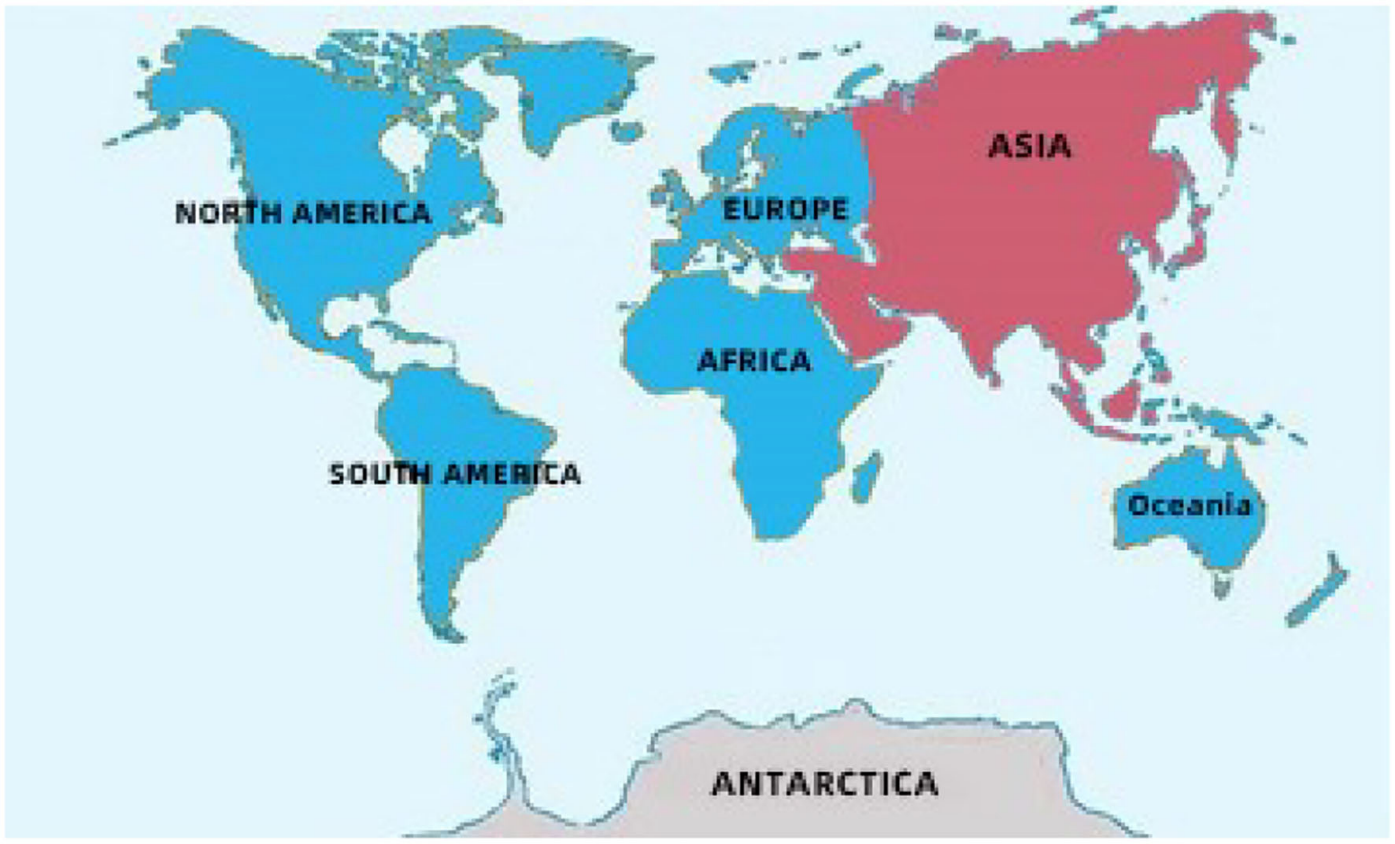
The main geographical distribution of kinds of vinegar in the world. Blue: The main producing areas of fruit kinds of vinegar. Red: The main producing areas of cereal kinds of vinegar.

Fruit vinegar is usually produced and used as seasonings in Europe, America, and Africa (Table 1 and Figure 1) (Solieri and Giudici, 2009; Matsushita et al., 2016; Sengun, 2017). Various fruits, such as grape, apple, pineapple, mango, jujube, and banana, are utilized to

**Table 1:** Some kinds of vinegar in the world.

**Vinegars**	**Raw materials**	**Vinegar names**	**Main producing areas**	**References**
Fruit vinegars	Grape	Balsamic vinegar (BV)	Italy	Morales et al., 2001; Liu and He, 2009; Solieri and Giudici, 2009; Cirlini et al., 2011; Hindi et al., 2016; Adebayo-Oyetoro et al., 2017; Hafpzan et al., 2017; Roda et al., 2017; Boonsupa et al., 2019; Xu et al., 2022;
		Sherry vinegar (SV)	Spain	
		Oxos vinegar	Greece	
		Raisin vinegar Wine vinegar	Middle East and USA Europe	
	Apple	Cider vinegar (CV)	Europe, USA and Canada	
	Pineapple	Pineapple vinegar (PV)	Africa and Taiwan (China)	
	Mango	Mango vinegar	Africa and Southeast Asia	
	Coconut	Coconut vinegar	Africa and Philippines	
	Plum	Plum vinegar	Africa, China and Japan	
	Banana	Banana vinegar	Africa	
	Date	Date vinegar	Africa and China	
Cereal vinegars	Rice	Japanese rice vinegar (JRV)	Japan	Nanda et al., 2001; Solieri and Giudici, 2009; Chung et al., 2017; Mudura et al., 2018
		Korean rice vinegar (KRV) Beijing rice vinegar (BRV)	Korea China	
		Zhenjiang aromatic vinegar (ZAV) Fujian *Monascus* vinegar	China China	
	Sorghum Wheat bran Barley Malt	Shanxi aged vinegar (SAV)	China	
		Sichuan bran vinegar (SBV)	China	
		Beer vinegar	Germany and Austria	
		Malt vinegar Distilled malt vinegar	Northern Europe and USA Northern Europe and USA	

produce the fruit vinegar (Chang et al., 2005). There are numerous famous traditional fruit vinegars in the world, such as traditional balsamic vinegar (TBV) and balsamic vinegar (BV) from Italy and Sherry vinegar (SV) from Spain, each of which is a protected geographical indication (PGI) product in Europe (Marrufo-Curtido et al., 2012). According to the historical documents, the fruit vinegar is considered to have firstly been invented, produced, and used by Egyptians, Sumerians, and Babylonians (Solieri and Giudici, 2009; Matsushita et al., 2016; Sengun, 2017). Many fruit vinegar production methods used in ancient times have been available still now. Normally, the traditional fruit vinegar is made *via* a submerged fermentation process, mainly including alcoholic and acetous fermentation processes (Figure 2) (Giudici et al., 2015). In the traditional process of fruit vinegar, the fruits are firstly crushed to obtain juice or must followed by cooking, which can improve the sugar concentration to avoid pollution by harmful microorganisms. Subsequently, alcohol and acetous fermentation, and vinegar aging occur almost simultaneously in wooden barrels, which is a spontaneous fermentation process driven by diverse microbes from materials and environments. Conversely, in the modern process of fruit vinegar, the alcoholization, acetification, and aging processes are successively carried out through, respectively, inoculating *Saccharomyces cerevisiae* and acetic acid bacteria (AAB) in different containers (Figure 2) (Giudici et al., 2015; Luzón-Quintana et al., 2021). During the production processes of the fruit vinegar, VVOCs are derived from the related raw materials, microorganisms, and chemical reactions, such as esterification reaction between acids and alcohols, Maillard reaction, and so on (Marín et al., 2002; Corsini et al., 2019). In addition to seasoning foods, the fruit vinegar was applied to treat wound inflammations, coughs, ulcers, and infectious diseases in ancient times (Chen et al., 2016).

**Figure 2:**
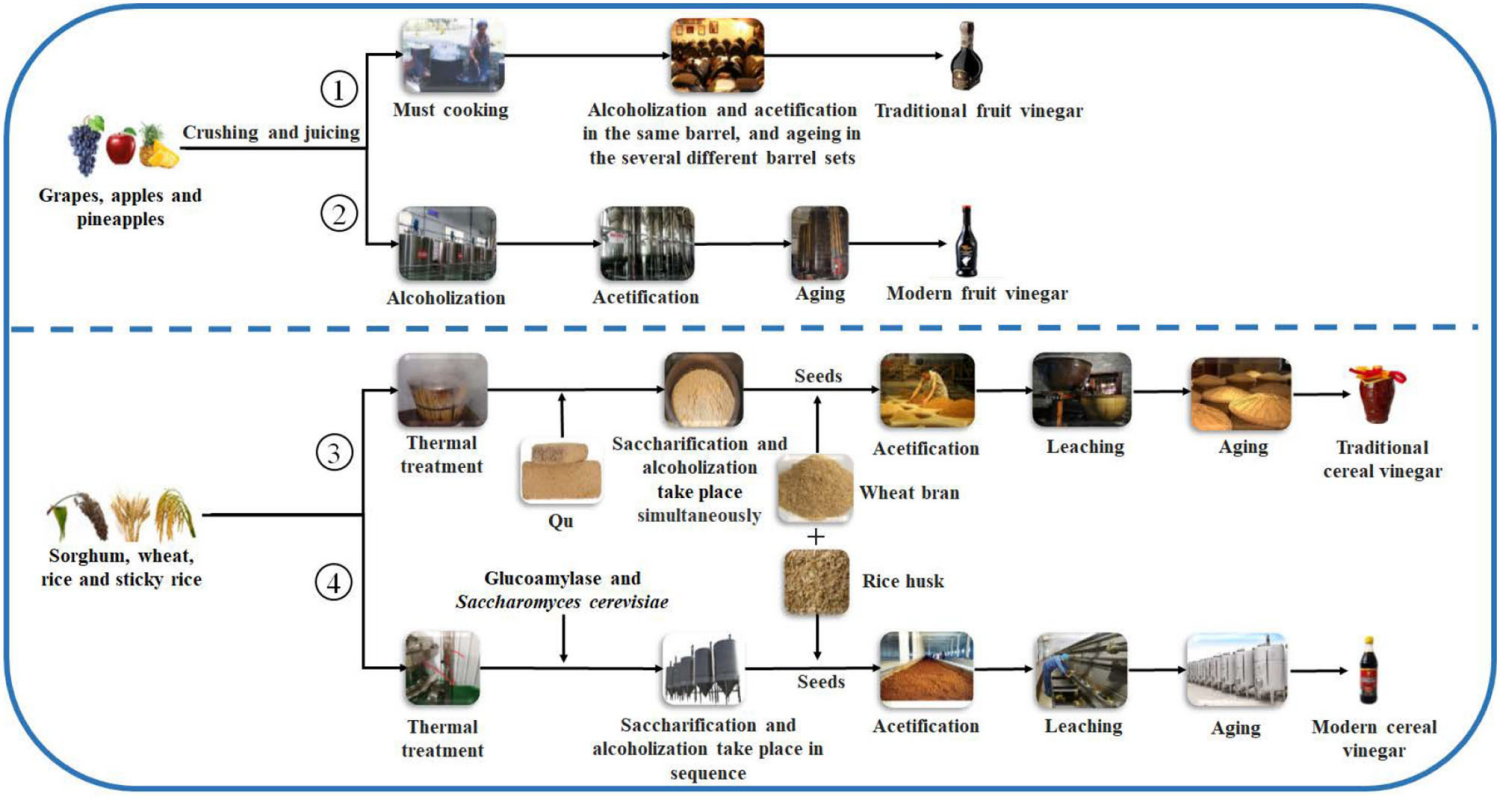
General schematic diagrams of vinegar brewing. ①: Traditional fruit kinds of vinegar brewed by liquid-state fermentation; ②: Modern fruit kinds of vinegar brewed by liquid state-fermentation; ③: Traditional cereal kinds of vinegar brewed by solid-state fermentation; ④: Modern cereal kinds of vinegar brewed by liquid- and solid-state co-fermentation.

### Cereal Vinegar

Cereal vinegar originated around 1,000 B.C. in China and now it is the most commonly appeared vinegar in China, Japan, Korea, and other Asian countries (Table 1 and Figure 1) (Chen et al., 2009). Cereal vinegar is often applied as a seasoning for various foods, such as boiled and steamed fish diets, sushi, seaweed salad, and so on (Solieri and Giudici, 2009; Matsushita et al., 2016; Sengun, 2017). The raw materials of the cereal vinegar mainly include sorghum, rice, wheat, corn, barley, and other materials rich in starch (Solieri and Giudici, 2009; Matsushita et al., 2016; Sengun, 2017). Traditional cereal vinegar is often prepared by a solid-state fermentation process (Figure 2). After the starchy materials, such as sorghum, rice, and wheat, are ground into pieces and steamed with vapors, the cooled steamed raw materials are mixed with Qu (koji, a kind of starter) powder, and saccharification and alcoholization start to carry out in a ceramic urn almost at the same time, respectively, by molds and their hydrolases, and yeasts from Qu. After that, rice husk or wheat bran and the vinegar “seeds” (a part of the last acetous fermented pastes) are put into the urn and mixed well to initiate the acetous fermentation. The purpose of adding rice husk or wheat bran is to make air (oxygen) more easily enter the acetous fermentation paste to promote the production of acetic acid by AAB. Finally, the fresh vinegar is obtained *via* leaching and aged in the ceramic urn. Compared to the traditional brewing method, alcoholic fermentation of modern cereal vinegar is usually carried out in a fermenter *via* a liquid-state fermentation process, during which saccharification and alcoholization are took place in sequence by adding glucoamylase preparation and *S. cerevisiae*. Then acetification begins in an oblong cement pool under solid-state fermentation after the alcoholized mash is mixed with rice husk or wheat bran and AAB. At last, the fresh vinegar obtained by leaching is aged in a tank (Chen et al., 2009).

There are some well-known cereal vinegars (Table 1 and Figure 1) in Asia, such as the Japanese rice vinegar (JRV), Korean rice vinegar (KRV), and four famous traditional cereal vinegars from China, namely, Shanxi aged vinegar (SAV), Zhenjiang aromatic vinegar (ZAV), Sichuan bran vinegar (SBV) and Fujian *Monascus* vinegar, each of which is brewed from the special cereal materials by the distinctive process, and possesses the unique aromas and flavors, although their brewing processes are basically the same (Liu et al., 2004; Chen et al., 2009; Cirlini et al., 2011; Zhou et al., 2017; Al-Dalai et al., 2019a, 2020a). Besides condiments, cereal vinegar, especially traditional ones, has been utilized to adjust blood pressure and prevent epidemic diseases since the 17th century in China (Chen et al., 2016; Lu et al., 2016). The cereal vinegar has also been reported to prevent the angiotensin-converting enzyme activity in spontaneously hypertensive rats (Nishikawa et al., 2001) and possesses anti-hypertensive and anti-inflammatory abilities (Murooka and Yamshita, 2008).

## Analytical Methods for the Vinegar Volatile Organic Compounds

VVOCs, which endow vinegar with unique aroma and flavor characteristics, are useful for the differentiation of various vinegars, especially PGI products (Solieri and Giudici, 2009; Chen et al., 2016; Matsushita et al., 2016; Sengun, 2017). VVOCs also play a great contribution to the vinegar qualities approved by consumers (Capone et al., 2013; Poinot et al., 2013; Wang et al., 2017).

Since VVOCs are volatile, gas chromatography (GC) and its derived methods, including GC-mass spectrometry (GC-MS), headspace solid-phase micro-extraction coupled with GC-MS (HS-SPME-GC-MS), and GC-olfactometry-MS (GC-O-MS), are the most commonly used methods to decipher VVOCs (Zhou et al., 2017; Al-Dalai et al., 2020a). Of course, high-performance liquid chromatography (HPLC) and dispersive liquid-liquid micro-extraction coupled with HPLC (DLLME-HPLC) are also effective methods to identify VVOCs, especially those VVOCs with poor volatility (Wang et al., 2017; Zhou et al., 2017).

### GC and Its Derived Methods

The theoretical basis of GC was proposed and developed by Anthony and Archer (Martin and Synge, 1941). Now GC is a kind of chromatography commonly used to separate and identify chemical compounds which can be vaporized without disintegration (Bartle and Myers, 2002). GC analytical abilities mainly depend on the boiling points of the analyzed compounds, their binding abilities with other compounds, and the separation abilities of the chromatographic columns (Welthagen et al., 2003). However, the boiling point of the analyte is not fixed, which is related to its niches, that is, closely related to the binding abilities between the separated component and other components in the sample (Bartle and Myers, 2002). Moreover, it is necessary to use the expensive standard compounds when VVOCs are analyzed by GC, leading to high costs. Recently, GC has rarely been used to analyze VVOCs.

GC-MS is one of the most commonly used techniques for qualitative and quantitative analysis of volatile substances from food, feed, and environmental samples since its infancy in 1955 (Zhao et al., 2018). GC-MS can easily detect and identify volatile compounds by comparing their fragments with those of standard substances from the public GC-MS databases (Zhao et al., 2018). Chinnici et al. (2009) used GC-MS to analyze VVOCs in TBV, BV, and SV and identified 90 VVOCs. Truta et al. (2010) also applied GC-MS to identify 23 VVOCs in CV.

Extraction methods also have an important impact on the detection of VVOCs in the GC-MS process, among which HS-SPME possesses the advantages of simple operation, high sensitivity as well as small reagent usage, and sample requirement (Xu et al., 2016). Therefore, HS-SPME coupled with GC-MS (HS-SPME-GC-MS) is developed and applied to analyze VVOCs due to its power, sensitiveness, and rapidness (Guerrero et al., 2007; Acena et al., 2011; Yu et al., 2012; Al-Dalai et al., 2019a). With HS-SPME, VVOCs are easily absorbed onto an absorbent coated fused silica fiber, and then dissociated into the GC injection port (Isidorov et al., 2003). The advantages of HS-SPME-GC-MS are simple, fast, accurate, sensitive, and linear in the analysis of VVOCs (Al-Dalai et al., 2019a,b). Nowadays, HS-SPME-GC-MS has become a convenient and efficient method to identify VVOCs (Griglione et al., 2015). Zhu et al. (2016a) employed an HS-SPME-GS-MS method with a satisfactory, repeatability, accuracy, and linearity to investigate the dissimilarity and similarity of different SAV samples. Roda et al. (2017) applied HS-SPME-GC-MS to determine VVOCs in the CV sample.

There are many kinds of VVOCs, and the contribution of different sorts of VVOCs to vinegar aroma and flavor profiles is also different. However, those VVOCs with high odor values but the low concentration level may not be detected by GC-MS (Song and Liu, 2018). So GC-O, a technique combining an olfactometer with GC, is utilized to identify VVOCs and analyze their odor activity values (Górecki et al., 1999; Al-Dalai et al., 2019a,b). Corsini et al. (2019) applied GC-O to determine odor-active compounds in TBV and found that the odor compounds in TBV were grouped into 7 categories. Since GC-O is not efficient for the qualitative analysis due to its low sensitivity, GC-O-MS, namely GC-O coupled with MS, is applied to quantify and identify VVOCs (Song and Liu, 2018). A total of 68 aroma and flavor compounds including 27 odorants were identified, and the higher caramel-like and buttery odor intensities were observed in aged ZAV compared with fresh ones (Al-Dalai et al., 2020b).

Sometimes single GC column is very difficult to fully separate target compounds in highly complex samples due to co-elution of target and non-target compounds, and an insufficient peak capacity (Zhu et al., 2016b). Therefore, the two-dimensional GC (GC × GC) combined with time-of-flight mass spectrometry (TOF-MS) is developed to efficiently separate and identify the target substances in complex samples *via* the separation of two GC columns (Castillo et al., 2011). Recently, Zhou et al. (2017) used HS-SPME coupled with GC × GC and TOF-MS (HS-SPME-GC × GC-TOF-MS) to investigate VVOCs in ZAV and found a total of 360 VVOCs based on mass spectrum match factors, structured chromatogram, and linear retention indices comparison, and methanethiol, 2-methyl-propanal, 2-methyl-butanal, 3-methyl-butanal, octanal, 1-octen-3-one, dimethyl trisulfide, trimethyl-pyrazine, acetic acid, 3-(methylthio)-propanal, furfural, benzene-acetaldehyde, 3-methyl-butanoic acid/2-methyl-butanoic acid, and phenethyl acetate were considered to be the main odorants, about half of which were identified as significant aroma constituents in ZAV for the first time. Moreover, three odor regions including more than one VVOC were found, since these odor regions were co-eluted in one dimensional GC, but they were well separated in the second axis of GC × GC analysis.

The advantages and disadvantages of GC and its derived methods are summarized in Table 2.

**Table 2:** Advantages and disadvantages of GC, HPLC, and their derivative methods to analyze VVOCs.

**Methods**		**Advantages**	**Disadvantages**	**References**
GC and its derivative methods	GC	Simply, sensitively and efficiently separate and detect VVOCs.	High cost when using expensive standard, and not suitable to determinate compounds with poor thermal stability	Murray, 2001; Bartle and Myers, 2002; Marín et al., 2002; Yu et al., 2012; Chen et al., 2013a; Chauhan et al., 2014; Zhou et al., 2017, 2020; Song and Liu, 2018
	GC-MS	Qualitatively and accurately describe the VVOC profiles from the different vinegar samples according to robust available GC-MS databases.	Contribution of each VVOC to the overall aroma and flavor of vinegar and the characteristic aroma-active and odor-active compounds cannot be detected.	
	SPME-GC-MS	Automatically absorb and concentrate VVOCs, avoid loss and pollution of VVOCs in sample pretreatment, and cut down sample preparation time, solvent usage and disposal costs.	Some VVOCs, such as acetoin, cannot be accurately detected owing to that the fibers are not sensitive to them.	
	GC-O	Directly determine key aroma active VVOCs, especially those with high odor intensities but the low concentration *via* the olfaction systems, such as the human olfactory or the olfactometric detectors.	Not efficient for the qualitative analysis of VVOCs.	
	GC-O-MS	Simultaneous detection of aroma-active VVOCs and acquisition of their corresponding chemical structural information due to combination of MS and olfactometric detector.	The working conditions may AFPfect the synchronization of the retention time of VVOCs in MS and the olfactometric detectors.	
	HS-SPME-GC x GC-TOMS	A very powerful and effective technique to analyze VVOCs due to high resolution, high sensitivity, high peak capacity and structured chromatograms, and especially qualitatively differentiate ions with similar m/z.	The standard mass spectrometry library is not rich enough, and it needs better and faster data processing software to treat huge data produced by HS-SPME-GC x GC-MS. High costs and careful maintenance are need.	
HPLC and its derivative methods	HPLC	A rapid, easy and precise separative and quantitative method for VVOCs with poor volatility and poor thermal stability.	Low sensitive and not suitable for some VVOCs.	Theobald et al., 1998; Yan and Wang, 2013; Wu et al., 2019
	DLLME-HPLC	A method with good sensitiveness, accuracy, short process and small wastes.	The extract solvents, such as chlorobenzene, chloroform, carbon tetrachloride and carbon disulfide, are toxic and harmful to the environment.	

### HPLC and DLLME-HPLC

HPLC is an analytic technology, which has the characteristics of fast analysis, and high separation efficiency for non-volatile compounds, such as amino acids, carbohydrates, and so on in samples (Das et al., 2014). HPLC also plays a significant role to analyze VVOCs (Langos et al., 2013). For example, Gaspar and Lucena (2009) applied HPLC to determine furfuraldehyde, 5-methylfurfural, and 5-hydroxymethylfurfural in BV, and Chen et al. (2010) detected acetoin and tetramethylpyrazine in traditional Chinese cereal vinegar by HPLC.

In order to reduce the sample preparation time of HPLC, DLLME is introduced for the pre-treatment of samples to enrich the target components, accelerate the detection process, and reduce the waste generated during the HPLC process (Berijani et al., 2006; Leong and Huang, 2008; Farajzadeh et al., 2009). In the DLLME process, a ternary solvent system comprising extraction and dispersion solvents is used, which can accelerate the dispersion of an organic extraction solvent into an aqueous sample to achieve highly efficient extraction (Yan and Wang, 2013; Russo et al., 2014; Shrivas and Kapadia, 2015; Bahadir et al., 2016), therefore, DLLME coupled with HPLC (DLLME-HPLC) is a sensitive, stable, accurate, rapid and inexpensive method to analyze VVOCs with poor volatilities. Wu et al. (2019) used DLLME-HPLC to rapidly determine tetramethyl-pyrazine in vinegar, indicating that this method is sensitive, stable, and accurate. The advantages and disadvantages of HPLC and DLLME-HPLC are listed in Table 2.

## Main VOCs from Vinegar

**Figure 3:**
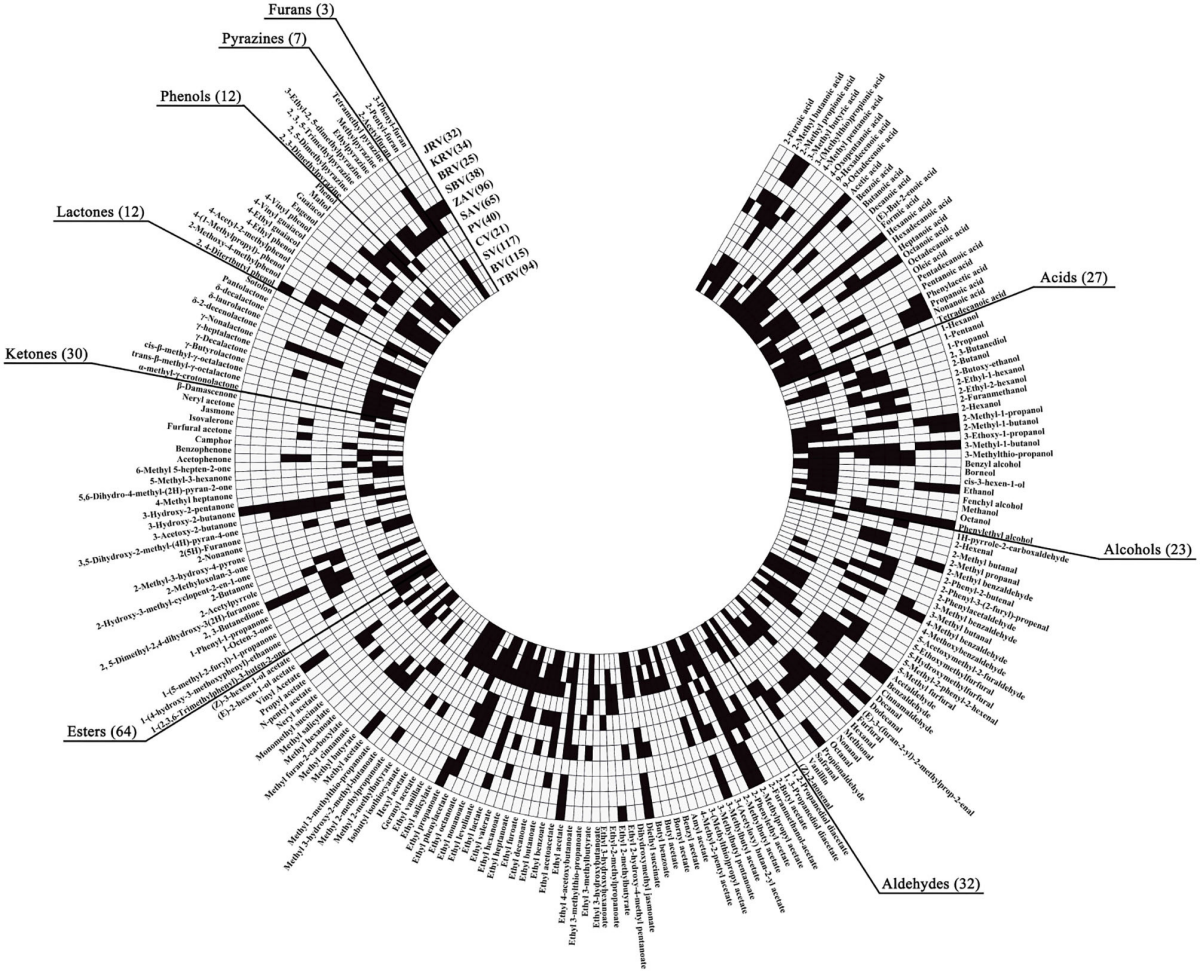
Profiles of the VOCs from different kinds of vinegar. The small black squares indicate that the VVOCs have been detected and reported in the corresponding kinds of vinegar, and the small white squares indicate that the VVOCs have not been determined and reported in the corresponding kinds of vinegar. BV, Balsamic vinegar; BRV, Beijing rice vinegar; CV, Cider vinegar; JRV, Japanese rice vinegar; KRV, Korean brown rice vinegar; PV, Pineapple vinegar; SAV, Shanxi aged vinegar; SBV, Sichuan bran vinegar; SV, Sherry vinegar; TBV, Traditional balsamic vinegar; ZAV, Zhenjiang aromatic vinegar.

**Figure 4:**
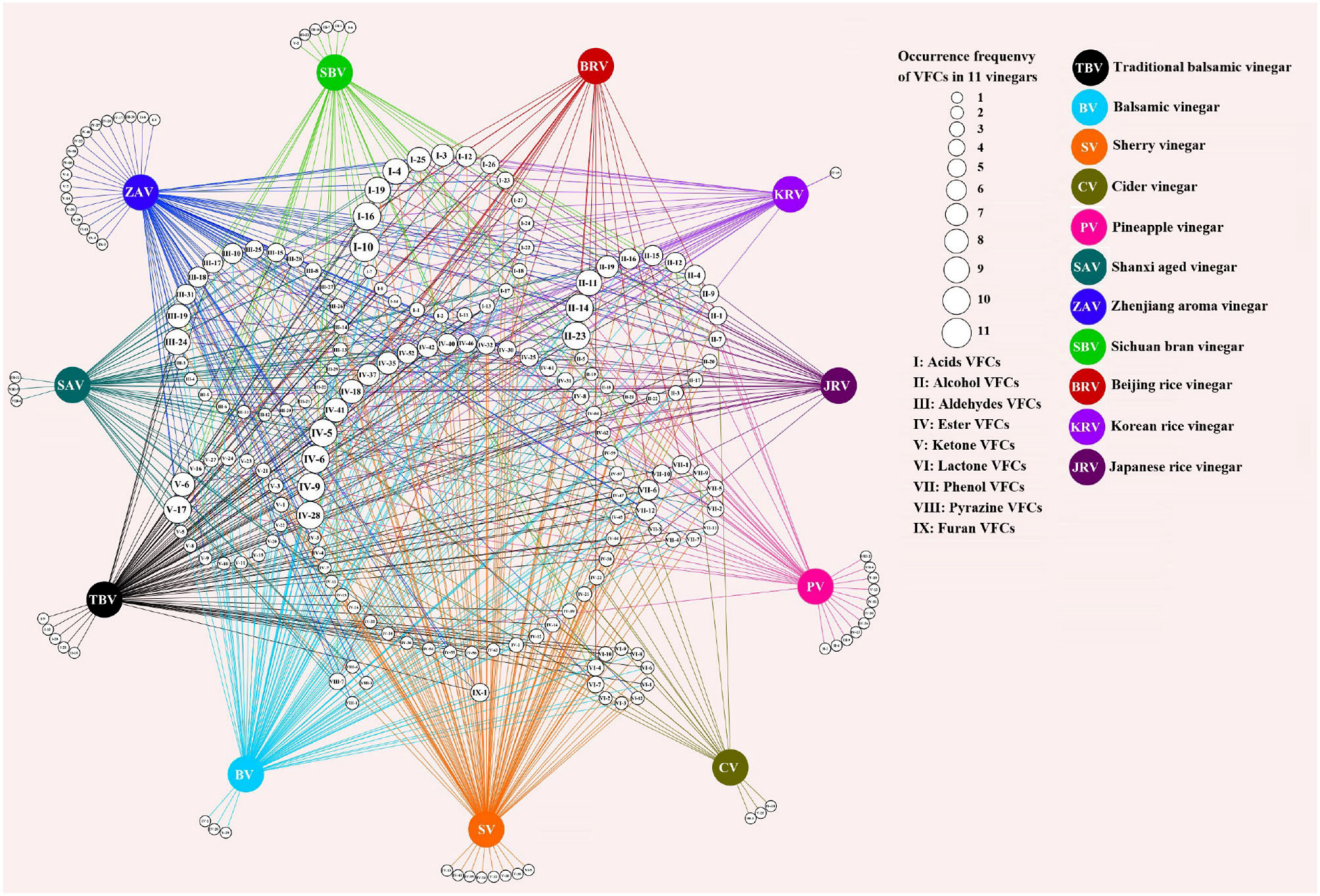
The shared and unique VOCs from different kinds of vinegar. The numbers in the small white circles in the middle represent the kinds of vinegar volatile organic components (VVOCs) (Annex Table 3) shared in different kinds of vinegar, and the sizes of the circles indicate the occurrence frequencies of VVOCs, and the larger the circle, the higher the frequencies of VVOCs in kinds of vinegar; the colorful circles represent the different kinds of vinegar; the numbers in the circles on the outside of the different kinds of vinegar express the unique VVOCs of the corresponding kinds of vinegar. The figure was drawn *via* Cytoscape v.3.7.2 (Shannon et al., 2003).

Both fruit and cereal vinegars, especially traditional ones, possess the characteristic aromas and flavors mainly given by different VVOCs. Herein we collect a total of 160 (Annex Table 1) and 124 (Annex Table 2) reported VVOCs from 5 fruit vinegars (TBV, BV, SV, CV, and PV) and 6 cereal vinegars (SAV, ZAV, SBV, Beijing rice vinegar (BRV), KRV, and JRV), respectively. Based on the chemical characteristics, VVOCs can be divided into 9 groups, which are acid, alcohol, aldehyde, ester, ketone, lactone, phenol, pyazine, and furan (Table 3). Although the total number of VVOCs reported in fruit (160) and cereal vinegar (124) is not too much different, the VVOCs' numbers of each VVOCs' group are quite different. The numbers of acid, alcohol, aldehyde, ester, ketone, lactone, phenol, pyazine, and furan VVOCs in fruit vinegar are 25, 22, 17, 53, 20, 10, 11, 1, and 1, respectively, while their numbers are 15, 14, 29, 32, 13, 4, 8, 6 and 3 in cereal vinegar, respectively (Table 3). Moreover, there is a great difference in the VVOC profile for each vinegar, including types and numbers of VVOCs (Figure 3 and Annex Table 3). For example, the number of VVOCs reported in SV is the largest, up to 117 species, while the number of VVOCs reported in CV is the least, only 21 species (Figure 3 and Annex Table 3). The common and unique VVOCs from different kinds of vinegar are shown in Figure 4.

**Table 3:** Classification of VOCs in fruit and cereal vinegars.

**VOC groups**	**VOC numbers**	
	**Fruit vinegars**	**Cereal vinegars**
Acids	25	15
Alcohols	22	14
Aldehydes	17	29
Esters	53	32
Ketones	20	13
Lactones	10	4
Phenols	11	8
Pyrazines	1	6
Furans	1	3
Total	160	124

### The Common and Unique VOCs in Fruit and Cereal Vinegar

Only 5 and 4 commonly appeared VVOCs (Table 4) are reported in 5 fruit vinegars (TBV, BV, SV, CV, and PV) and 6 cereal vinegars (SAV, ZAV, SBV, BRV, KRV, and JRV), respectively; And a total of 30 and 28 unique ones are found from 5 fruit vinegars and 6 cereal vinegars, respectively (Figure 4).

**Table 4:** The commonly appeared VOCs in fruit and cereal vinegars.

**Volatile organic components**	**Fruit vinegars**					**Cereal vinegars**				
	**TBV**	**BV**	**SV**	**CV**	**PV**	**SAV**	**ZAV**	**SBV**	**BRV**	**KRV**	**JRV**
**Acids (2)**
Acetic acid	+	+	+	+	+	+	+	+	+	+	+
Hexanoic acid	+	+	+	nd	+	+	+	+	+	+	+
**Alcohols (2)**
3-Methyl-1-butanol	+	+	+	+	+	+	+	nd	+	+	+
Phenylethyl alcohol	+	+	+	nd	+	+	+	+	+	+	+
**Esters (4)**
2-Methylpropyl acetate	+	+	+	+	+	+	+	nd	+	+	+
Ethyl acetate	+	+	+	+	+	+	+	nd	+	+	+
Ethyl hexanoate	+	+	+	+	+	nd	+	nd	+	nd	nd
Phenylethyl acetate	+	+	+	nd	+	+	+	+	+	+	+

*BV, Balsamic vinegar; BRV, Beijing rice vinegar; CV, Cider vinegar; JRV, Japanese rice vinegar; KRV, Korean brown rice vinegar; PV, Pineapple vinegar; SAV, Shanxi aged vinegar; SBV, Sichuan bran vinegar; SV, Sherry vinegar; TBV, Traditional balsamic vinegar; ZAV, Zhenjiang aromatic vinegar. +, Detected; nd, Not detected. The numbers in brackets are the VVOCs numbers*.

The results from Figure 4 and Table 4 have revealed that both fruit and cereal vinegars possess more unique VVOCs and fewer shared VVOCs, which may be the reason why different kinds of vinegar have unique characteristics. There is a total of 30 unique VVOCs including 5, 3, 8, 3, and 11 unique ones (Figure 4) from TBV, BV, SV, CV, and PV, respectively, while there is a total of 28 unique VVOCs containing 3, 18, 6, 0, 1, and 0 unique ones in SAV, ZAV, SBV, BRV, KRV, and JRV, respectively (Figure 4). Only 5 VVOCs simultaneously appear in all 5 fruit vinegars, which are 1 acid VVOC, acetic acid; 1 alcohol VVOC, 3-methyl-1-butanol; 3 ester VVOCs, including 2-methyl-propyl acetate, ethyl acetate, and ethyl hexanoate, while only 4 common VVOCs co-existed in 6 cereal vinegars, which contains 2 acid VVOCs, acetic acid, and hexanoic acid; 1 alcohol VVOC, phenylethyl alcohol; 1 ester VVOC, phenylethyl acetate (Table 4).

In regard to the commonly appeared VVOCs, acetic acid as a domain VVOC, also the main constituent and a precursor of acetyl ester VVOCs, exists in all fruit and cereal vinegars (Table 4). Moreover, except ethyl hexanoate, 3 common VVOCs, including 3-methyl-1-butanol, 2-methyl-propyl acetate, and ethyl acetate from fruit vinegars (Table 4), also appear in five (BRV, JRV, KRV, SAV, and ZAV) of the six kinds of cereal vinegars (Annex Table 3), while 3 common VVOCs containing hexanoic acid, phenylethyl alcohol and phenylethyl acetate from cereal vinegars (Table 4) exist in four (BV, PV, SV, and TBV) of the five kinds of fruit vinegars as well (Annex Table 3). Consequently, besides acetic acid, the above-mentioned shared VVOCs from both fruit and cereal vinegars, respectively, might also be considered the common VVOCs to a large extent.

### Common and Unique VOCs Among Pairwise Kinds of Vinegar

Besides simultaneously appearing VVOCs in both fruit and cereal vinegars (Table 4), the common and unique VVOCs between the two kinds of vinegar are listed in Table 5.

**Table 5:** Common and unique VOCs between each fruit vinegar and cereal vinegar.

	**Fruit vinegars**					**Cereal vinegars**				
	**TBV**	**BV**	**SV**	**CV**	**PV**	**SAV**	**ZAV**	**SBV**	**BRV**	**KRV**	**JRV**
TBV	_	84/31,9	78/39,15	10/11,83	22/18,71	27/38,66	36/60,57	15/23,78	18/7,75	23/11,70	22/10,71
BV	84/9,31*	_	97/20,18	15/6,100	23/17,92	40/25,75	50/46,65	23/15,92	19/6,96	28/6,87	26/6,89
SV	78/15,39	97/18,20	_	15/6,102	27/13,90	41/24,76	52/44,65	23/15,94	21/4,96	28/6,89	26/6,91
CV	10/83,11	15/100,6	15/102,6	_	7/33,14	8/57,13	10/86,11	1/37,20	7/18,14	9/25,12	10/22,11
PV	22/71,18	23/92,17	27/90,13	7/14,33	_	19/46,21	24/72,16	9/29,31	15/10,25	14/20,26	13/19,27
SAV	27/66,38	40/75,25	41/76,24	8/13,57	19/21,46	_	58/38,7	23/15,42	21/4,44	24/10,41	20/12,45
ZAV	36/57,60	50/65,46	52/65,44	10/11,86	24/16,72	58/7,38	_	27/11,69	23/2,73	25/9,71	21/11,75
SBV	15/78,23	23/92,15	23/94,15	1/20,37	9/31,29	23/42,15	27/69,11	_	12/13,84	11/23,85	9/23,87
BRV	18/75,7	19/96,6	21/96,4	7/14,18	15/25,10	21/44,4	23/73,2	12/84,13	_	16/18,9	16/16,9
KRV	23/70,11	28/87,6	28/89,6	9/12,25	14/26,20	24/41,10	25/71,9	11/85,23	16/9,18	_	29/3,5
JRV	22/71,10	26/89,6	26/91,6	10/11,22	13/27,19	20/45,12	21/75,11	9/87,23	16/9,16	29/5,3	_

*BV, Balsamic vinegar; BRV, Beijing rice vinegar; CV, Cider vinegar; JRV, Japanese rice vinegar; KRV, Korean brown rice vinegar; PV, Pineapple vinegar; SAV, Shanxi aged vinegar; SBV, Sichuan bran vinegar; SV, Sherry vinegar; TBV, Traditional balsamic vinegar; ZAV, Zhenjiang aromatic vinegar*.

* *Shared VVOCs/unique VVOCs_(TBV)_, unique VVOCs_(BV)_*.

The results in Table 5 show that the number of shared VOCs is generally lower than those of unique VOCs in both fruit and cereal vinegars. For instance, between TBV (fruit vinegar) and SAV (cereal vinegar), the number of their common VVOCs is only 27, but the numbers of their unique VVOCs are 66 and 38, respectively.

With regard to the common VVOCs between fruit and cereal vinegars, the main raw materials of vinegar may have a significant impact on the common VVOCs among different kinds of vinegar. For example, 3 European PGI fruit vinegar products TBV, BV, and SV, all of which are made from grapes (Table 1), possess 84, 78, and 97 common VVOCs between TBV and BV, TBV and SV, and SV and BV, respectively (Table 5), while the common VVOCs between TBV and CV, TBV and PV made by grape, apple and pineapple (Table 1), respectively, are only 10 and 22. Similarly, except for 2 Chinese PGI cereal vinegar, SAV and ZAV have a high number of common VVOCs (58), the numbers of the common VVOCs among the other cereal vinegars are generally less than their unique VVOCs (Table 5), it may be that the main raw materials of SAZ, ZAV, SBV, BRV, KRV, and JRV are sorghum, glutinous rice, wheat bran, polished rice and unpolished rice, respectively (Table 1). In one word, the main raw materials of vinegar may greatly affect the common VVOCs between vinegar, which will be discussed in Section VVOCs From the Raw Materials.

In regard to unique VVOCs from fruit or cereal vinegar, in addition to coming from the main raw materials, they are also derived from the unique brewing processes. For instance, although the common VVOCs between TBV and BV, both of which are from Modena and Reggio Emilia areas of Italy, and made from grapes, reach up to 84 species, they still possess 9 and 31 unique VVOCs for TBV and BV, respectively. These unique VVOCs of TBV and BV may come from their different brewing processes since both TBV and BV have almost the same brewing processes, but at least the aging time of TBV is longer than that of BV (Consonni et al., 2008), which will be described in Section VVOCs From the Aging Process.

Besides the main raw materials and the unique processes of vinegar, other factors, such as the relative microorganism involved in vinegar brewing, may also affect VVOCs of vinegar, which will be described in detail in Section VVOCs Produced by Microorganisms.

## VVOC Formation Mechanisms

Vinegar are conspicuous by their unique aromas and flavors mainly from their raw materials, microbial communities, and process technologies (Morales et al., 2001; Solieri and Giudici, 2009; Matsushita et al., 2016; Sengun, 2017). Frankly, it is very difficult to exactly distinguish VVOCs where they are from, because some VVOCs may come alone from the raw materials, microorganisms, and processes, or from two or three of them. Moreover, VVOCs dynamically change during the whole production process of vinegar. In order to make readers more easily understand whether VVOCs come mainly from raw materials, microbes, or brewing technologies of vinegar, we will summarize and discuss in this section the effects of the raw materials, microbial population, or brewing technologies of vinegar on VVOCs, respectively.

### VVOCs From the Raw Materials

Although there is no relevant investigation on whether the components in raw materials can be directly transformed into VVOCs, some studies have shown that the raw materials have a great impact on VVOCs, and play significant roles for VVOCs (Marrufo-Curtido et al., 2012; Wang et al., 2012; Chung et al., 2017).

Marrufo-Curtido et al. (2012) used the stir bar sorptive extraction GC-MS method to analyze VVOCs from 2 Italian PGI vinegar, traditional balsamic vinegar of Modena (TBVM), balsamic vinegar of Modena (BVM) and found that their raw materials had a very significant influence on their VVOCs. Most of more than 100 VVOCs from TBVM brewed by the cooked grape must, and from BVM made by wine, were very similar, but some of these VVOCs, such as (E)-2-hexen-1-ol acetate, bornyl acetate, ethyl 2-phenyl acetate, methyl hexanoate, isobutyl isothiocyanate, 9-hexadecenoic acid, octadecanoic acid, 3-ethoxy-1-propanol, 4-acetyl-2-methylphenol I, 4-acetyl-2-methylphenol II, δ-selinene, citronellene, 2-acetyl-2, 5-dimethyl-furan, methyl styrene, tridecane, tetradecane and pentadecane, total 18 VVOCs, were detected only in TBVM, whereas the VVOCs, including ethyl propanoate, isovaleraldehyde, and butanoic acid, were found only in BVM.

Wang et al. (2012) applied tartary buckwheat as the main raw material to make SAV, which is normally brewed by sorghum (Table 1) and found that 45 VVOCs by GC-O-MS, among which 11 VVOCs, including dimethyl trisulfide, 2-methyl-2-butenal, benzene acetaldehyde, ethyl myristate, 1-pentanol, butanoic acid, 2-butanone, 2, 6-dimethylpyrazine, 2, 3-dimethylpyrazine, 2-ethyl-5-methylpyrazine and benzothiazole, have not been reported in other kinds of vinegar, including SAV made by sorghum.

Chung et al. (2017) evaluated VVOCs from 2 Chinese (C1 and C2) vinegar and 3 Japanese vinegar (J1–J3) with SPME-GC-MS to confirm the effects of their raw materials on VVOCs and discovered that VVOCs, including phenyl acetic acid, decanoic acid and ortho-tolualdehyde, were detected only in C2, not in C1, which were brewed by brown rice and purple rice, respectively, while VVOCs, such as vinyl acetate, propionic acid, phenyl acetic acid, para-tolualdehyde, and diacetyl, were found only in J1, not in J2, which were brewed by brown rice and polished rice, respectively.

### VVOCs Produced by Microorganisms

Microorganisms involved in vinegar production have a great influence on VVOCs, especially on traditional ones which are brewed by spontaneous fermentation (Solieri and Giudici, 2009; Matsushita et al., 2016; Sengun, 2017). Besides *S. cerevisiae* and AAB, there are lactic acid bacteria (LAB), other bacteria, and other yeasts involved in vinegar production (Solieri and Giudici, 2009; Li et al., 2015; Matsushita et al., 2016; Sengun, 2017). Moreover, for cereal vinegar, the filamentous fungi, such as *Aspergillus* spp., *Mucor* spp., *Rhizopus* spp., *Monascus* spp., *Alternaria* spp., and so on, are also involved in the starch decomposition (saccharification) during the cereal vinegar process (Chen et al., 2009; Li et al., 2015; Wang et al., 2016). These microorganisms can not only directly produce acid- and alcohol-like VVOCs, but also provide the substrates for other VVOCs formation in the process of vinegar production, especially in the processes of vinegar heating and aging, to produce various ester VVOCs *via* the esterification of acids and alcohols.

#### VVOCs by Microorganisms in the Alcoholic Fermentation

During the alcoholic fermentation process (AFP) of vinegar, many different microbial strains are isolated, especially *S. cerevisiae* found in all types of vinegar (Solieri and Giudici, 2009). Wang et al. (2016) reported that the dominant yeast species in AFP of fruit vinegar was *S. cerevisiae*. Rainieri and Zambonelli (2009) reported that the most commonly appearing yeast species was also *S. cerevisiae* since the high sugar environment conditions of AFP were more suitable for the growth of *S. cerevisiae* strains than that of other yeast species. Besides *S. cerevisiae*, non-*Saccharomyces*, such as *Candida* spp., *Cryptococcus* spp., and *Debaryomyces* spp., and bacteria, in particular, LAB commonly appeared in AFP, which also contribute greatly to VVOCs formation (Li et al., 2015; Wang et al., 2016).

For cereal vinegar, molds normally dominate at the initial stage of AFP, since the starch in cereal materials first needs to be digested into sugars by molds and their producing hydrolases, such as amylase and glucoamylase (Wang et al., 2016). Actually, saccharification and AFP are almost simultaneously carried out at the AFP initial stage of cereal vinegar (Zhao et al., 2018; Al-Dalai et al., 2019a, 2020a). Li et al. (2015) reported that the genera of *Aspergillus, Absidia, Mucor, Rhizopus, Saccharomyces*, and *Bacillus*, were the main microorganisms at the saccharification and AFP of cereal vinegar. And the genera of *Monascus, Penicillium, Trichoderma, Eurotium, Pichia, Hansenula, Lodderomyces, Rhodotorula, Zygosaccharomyces, Pediococcus, Weissella*, and *Clostridium* were also found in AFP of cereal vinegar, which may contribute to VVOCs production of cereal vinegar, too (Wang et al., 2016).

A large number of studies (Romero et al., 1986; Arrizon et al., 2006; Molina-Guerrero et al., 2007; Xiao et al., 2011; Nie et al., 2013; Jiang et al., 2019) have shown that microorganisms involved in the vinegar AFP have a great impact on VVOCs of fruit and cereal vinegar, especially on the alcohol-like VVOCs, mainly including ethanol, methanol, 1-propanol, 2-methyl propanol, n-butanol, 1-butanol, 2-methyl butanol, 3-methyl butanol, isobutanol, cis-3 hexanol, hexanol, heptanol, isoamylic alcohol, 2-phenyl ethanol, and acetoin. Some also have a significant influence on the ester VVOCs, such as ethyl hexanoate, ethyl octanoate, diethyl succinate, 2-phenylethyl acetate, isoamyl acetate and ethyl lactate, which are the main VVOCs in both of fruit and cereal vinegar. These alcohol- and ester-like VVOCs produced by microorganisms in AFP of vinegar make all kinds of vinegar appear pleasantly alcoholic floral, sweet and fruity odors (Romero et al., 1986; Molina-Guerrero et al., 2007).

Moreover, Han et al. (2001) discovered that the 3-methylbutanoic acid of VVOC was a byproduct of yeast metabolism. Various kinds of non-*Saccharomyces* species might positively shape the aroma and flavor profile of vinegar. For example, *C. stellata* can generate high concentrations of ethyl acetate, and acetoin in TBV (Ciani, 1998; Solieri and Giudici, 2008). In addition, *Lactobacillus* species present in AFP of cereal vinegar, including *Lactobacillus fermentum, Lactobacillus plantarum, Lactobacillus buchneri*, and *Lactobacillus casei*, can increase the contents of lactic acid in cereal vinegar, and modify their aromas and flavors (Chen et al., 2009; Wu et al., 2012; Li et al., 2015). Recently, Zhang et al. (2019) discovered that 2-phenyl ethanol of VVOC was derived from yeast metabolites *via* the reduction of phenyl-acetaldehyde during AFP.

#### VVOCs by Microorganisms in the Acetic Acid Fermentation

In the acetic acid fermentation process (AAFP) of vinegar, AAB, such as *Acetobacter* spp., *Komagataeibacter* spp., *Gluconobacter* spp., and *Gluconacetobacter* spp., are the absolutely dominant microorganisms, but other bacteria, mainly including *Lactobacillus* spp., *Pediococcus* spp., *Bacillus* spp., *Acinetobacter* spp., *Staphylococcus* spp., and *Pantoea* spp., are also involved in AAFP (Berry and Watson, 1987; Wang et al., 2016; Zhu et al., 2018; Jiang et al., 2019). These bacteria, especially AAB, can produce not only acidic VVOCs but also aldehyde and ketone VVOCs, which are also used as substrates to produce other VVOCs, such as ester-like VVOCs.

The strains from the genera of *Acetobacter* and *Komagataeibacter* are the main AAB strains, which are currently utilized in the production of fruit and cereal vinegar by liquid-state and solid-state fermentation, respectively (Matsushita et al., 2016). The acidic substances, such as acetic acid generated by strains of both AAB genera in AAFP, are the main VVOCs, and also the important precursors of ester-like VVOCs (Yu et al., 2012; Al-Dalai et al., 2019b). Besides, both AAB strains can also produce other VVOCs, such as acetoin, 3-acetoxy-2-butanone, and furfural (Zhu et al., 2018). The genus of *Lactobacillus* involved in AAFP can improve the contents of VVOCs, such as lactic acid, benzaldehyde, and acetaldehyde (Wu et al., 2017; Zhu et al., 2018). Wang et al. (2016) found that *Lactobacillus* spp. and *Gluconacetobacer* spp. largely contributed to the ester-like and heterocyclic VVOCs, such as isobutyl acetate, 3-hydroxy-2-butanone-acetate, ligustrazine, and *Staphylococcus* spp., were positively correlated for aldehyde-like VVOCs production during AAFP process of ZAV. The investigation conducted by Li et al. (2016) and Zhu et al. (2018) indicated that the genus of *Bacillus* was highly correlated with the acetate esters, and the genera of *Lactococcus, Pantoea, Pediococcus*, and *Rhizobium* could produce some ester-like VVOCs, including ethyl acetate, hexanoic acid ethyl ester, and propanoic acid 2-hydroxy-ethyl ester, during AAFP of SAV. Acetoin, a characteristic VVOC in cereal vinegar, and the precursor of pyrazine-like VVOCs, which is produced by AAB from acetolactate and 2, 3-butanediol (Zhao et al., 2018), is also yielded mainly in AAFP of cereal vinegar (Chen et al., 2013b; Al-Dalai et al., 2019b).

#### Formation Network of VVOCs Driven by Microbiota

In the production process of vinegar, especially traditional ones, besides microorganisms, such as *S. cerevisiae* and AAB, many other microbes, such as non-*Saccharomyces*, LAB, and molds, also participate in the vinegar production. These microbes can not only directly produce various VVOCs, but the VVOCs produced by one microorganism may also be applied as the substrates by another microorganism to transform into other VVOCs, resulting in forming very rich and complex VVOCs. In brief, the VVOC formation in the fermentation process of vinegar *via* microorganisms is very complex, dynamic, and networked.

**Figure 5:**
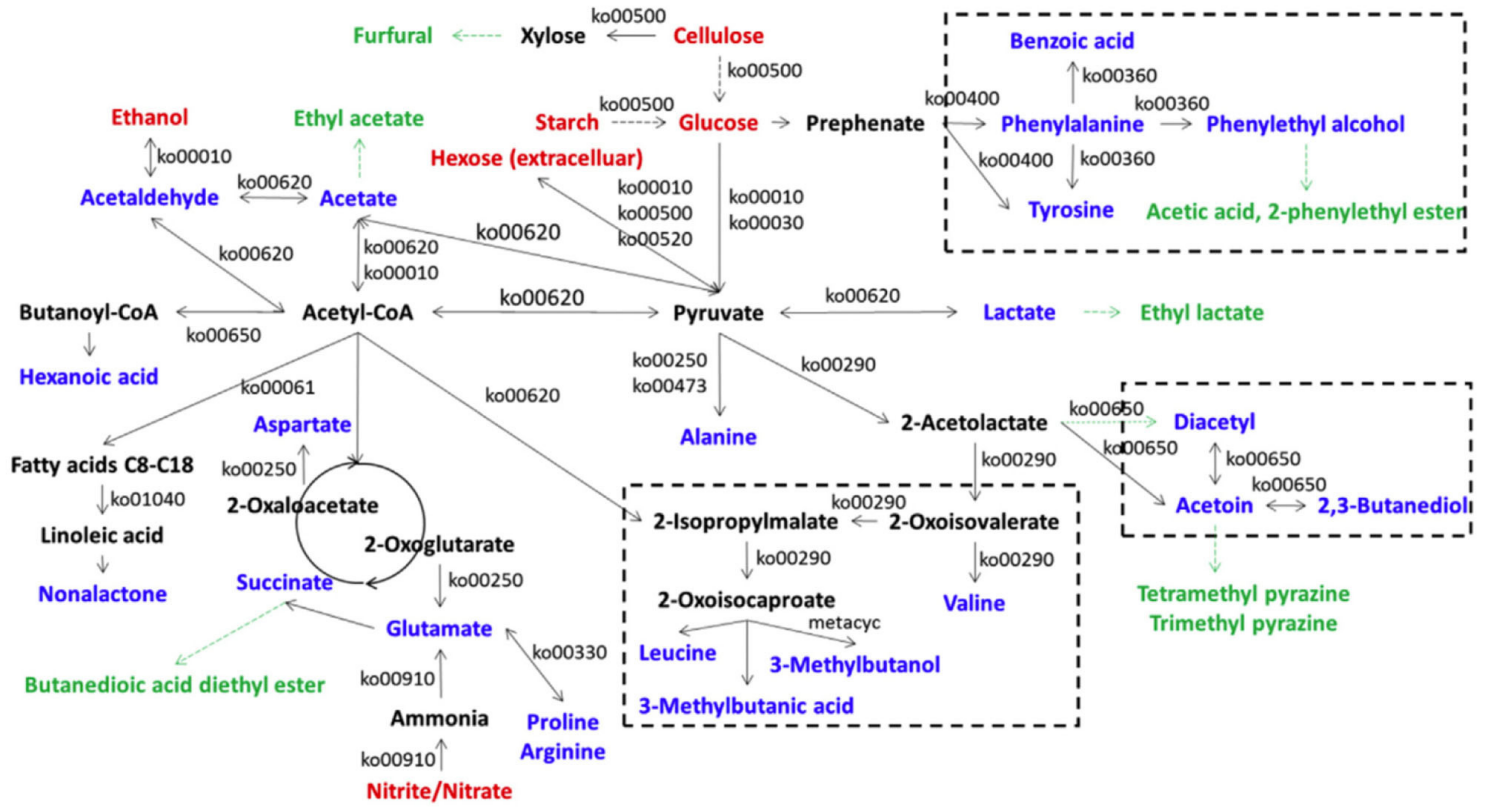
The metabolic network for substrate breakdown and formation of VOCs and amino acids *via* the microbial community in traditional Zhenjiang aged vinegar (Cited and modified from Wu et al., 2017). Red fonts represent the main substrates from the raw materials; deep black fonts represent amino acids or the intermediate compounds relative to the formation of VVOCs; black fonts represent the enzymes in the relative pathways predicted by the Kyoto Encyclopedia of Genes and Genomes; green fonts represent non-enzyme producing VVOCs.

Lu et al. (2016) found that *A. pasteurianus* G3-2, *L. brevis* 4-22, *L. fermentum* M10-3, and *L. buchneri* F2-5 were the main producers of acetoin from acetolactate in ZAV, and the acetoin concentrations in two groups of co-cultures of *L. brevis* 4-22 plus *A. pasteurianus* G3-2, and *L. fermentum* M10-3 plus *A. pasteurianus* G3-2 were obviously higher than those in monocultures of these LAB, while *L. buchneri* F2-5 did not produce more acetoin when co-cultured with *A. pasteurianus* G3-2. Wu et al. (2017) used the metagenomic approach to clarify the *in situ* metabolic network of key microbes responsible for VVOCs synthesis in traditional ZAV (TZAV) and discovered that the dominant characteristic VVOCs, such as acetoin, diacetyl, 3-methylbutanol, tetramethylpyrazine, 3-methylbutanoic acid, and 2, 3-butanediol, were mainly relative to the genera of *Lactobacillus* and *Acetobacter*, and the families of Mollicutes and Actinobacteria, which are involved in these VVOCs formation *via* the acetoin-diacetyl-2, 3-butanediol biosynthetic network. Besides, the production of 3-methylbutanol and 3-methylbutanoic acid of VVOCs in TZAV were relative to microorganisms from Euryarchaeota, Actinobacteria, Bacteroidetes, Elusimicrobia, Cyanobacteria, *Lactobacillus* spp., Bacillales, Clostridia, Synergistetes, Burkholderiales, *Acetobacter* spp., Proteobacteria, ε-Proteobacteria, and Rhodobacterales. Moreover, the authors predicted the metabolic network for substrate breakdown, and the formation of 23 VVOCs and 7 amino acids (alanine, arginine, aspartate, glutamate, phenylalanine, proline, and tyrosine) *via* the microbial community during the TZAV process (Figure 5), in which acetyl-CoA and pyruvate are considered as the core intermediate compounds.

### VVOCs From Vinegar's Unique Processes, Including Heating, Aging, and Others

Heating and aging treatments are two necessary and important processes for most vinegar products. Almost all vinegar products need to be heated to eliminate food-borne pathogens or other microorganisms, so as to ensure the food safety of vinegar before bottling and entering the markets. And most vinegar products, especially traditional ones, should be aged before entering the markets (Solieri and Giudici, 2009; Sengun, 2017). For instance, traditional SAV should be aged for at least 3 years in the open-top ceramic jar through the traditional folk technology called “exploring blazing sun in summer and taking out ice in winter” (Liang et al., 2016); TBV, a PGI vinegar, typically produced in Italian Reggio Emilia and Modena area should be kept in the casks made of different types of wood (oak, mulberry, chestnut, or juniper) for at least 12 years according to Italian laws (Giordano et al., 2003).

During heating and aging processes, components in vinegar can be concentrated and adjusted, and many VVOCs can be produced mainly through Maillard reaction, esterification reactions, and other reactions, such as degradation of amino acids (Lee and Shibamoto, 2002; Giordano et al., 2003; Cirlini et al., 2011; Wang et al., 2012; Liang et al., 2016; Zhou et al., 2020). For example, VVOCs of furan and its derivatives, which are characteristics of the caramel-like flavor of vinegar, is formed mainly *via* the Maillard reaction between reducing sugars and amino acids, which mainly occurs during the heating and aging processes of vinegar. The Maillard reaction usually produces N-glycosilamines and N-fructosylamines in the first step, then their isomerization forms 1-amino-1-deoxy-2-ketoses or 1-amino-2-deoxy-2-aldoses (Amadori compounds), which are the main precursors of furan-like VVOCs (Lee and Shibamoto, 2002).

Other vinegar brewing processes also have a great impact on VVOCs, in particular, VVOCs of traditional kinds of vinegar produced by modern methods often have a great difference compared with those by traditional methods, although their raw materials are the same (Zhao et al., 2018; Al-Dalai et al., 2019a, 2020a).

#### VVOCs From the Heating Process

In the SAV brewing process, there is a unique heating process in which about one-third of vinegar Pei (also called vinegar paste) is heated in the pottery jar (Chen et al., 2009). During the heating process, the vinegar Pei is turned over every day, and its temperature is gradually increased from 30°C to 90°C at the beginning of 3 days, and then gradually decreased to about 30°C in the following 2–3 days. The color of vinegar Pei becomes from yellow to dark brown after heating. Then, two-thirds of vinegar Pei without heated treatment is mixed with the heated vinegar Pei and drenched to obtain the final vinegar product (Chen et al., 2009). About 300 years ago, the heating vinegar Pei technology started to be applied in the SAV production, and at the beginning, the main purpose of heating vinegar Pei is to increase the color of SAV. Later, it happens that after heating, the SAV aroma and flavor are also improved. This heat-treated technology has been retained until now and has become a unique process to distinguish SAV from other cereal vinegar (Chen et al., 2009). During the heating process, many VVOCs in SAV are produced *via* the Maillard reaction and other associated reactions, such as the degradation of amino acids (Wang et al., 2012).

Wang et al. (2012) found that a total of 45 VVOCs were detected in SAV made by tartary buckwheat, and most of them were significantly increased during the first 3 days of the heating process (6 days at 85°C) and then decreased from the fourth day onward. They also found that 9 new VVOCs, including 2 alcohol ones, 2 aldehyde ones, 1 ketone one, and 4 pyrazine ones, have appeared, and 3 VVOCs, including 2 alcohol ones and 1 ester one, were disappeared in SAV treated after 6 days at 85°C (Table 6).

**Table 6:** Emerged and disappeared VOCs in SAV after heating 6 days at 85°C.

	**VVOCs**
Emerging VVOCs (9)	**Alcohols (2)**
	1-Pentanol
	Trimethyl oxazole
	**Aldehydes (2)**
	Furfural
	5-Methylfurfural
	**Ketone (1)**
	Acetophenon
	**Pyrazines (4)**
	2, 5-Dimethylpyrazine
	2, 6-Dimethylpyrazine
	2, 3-Dimethylpyrazine
	Trimethylpyrazin
Subsiding VVOCs (3)	**Alcoholes (2)**
	3-Butanediol
	Phenol
	**Ester (1)**
	Ethyl myristate

Giordano et al. (2003) reported that the contents of 2-furfural and 5-methylfurfural furfural (furan derivatives) in BVs, especially in TBVs, were much higher than those in other fruit vinegar of white and red wines, and these VVOCs of furan derivatives might result from Maillard reactions during cooking grape must of TBVs (Figure 2).

#### VVOCs From the Aging Process

Like heating treatment, the aging process of vinegar can also yield many VVOCs, especially those VVOCs related to Maillard reaction and esterification, such as furan-, pyrazine- and ester-like VVOCs. At the same time, vinegar will become softer and more concordant with higher odor intensity of caramel-like, buttery, and overall complexity after aging, and the quality, aroma, and flavor of vinegar are greatly improved (Zhou et al., 2017, 2020). Therefore, many traditional vinegar products, especially PGI ones, such as SAV from China and TBV from Italy, require a long period of aging (Giordano et al., 2003; Solieri and Giudici, 2009; Liang et al., 2016).

With regard to the aging effects on VVOCs in fruit vinegar, Marrufo-Curtido et al. (2012) used the stir bar sorptive extraction coupled with GC-MS to analyze VVOCs from 26 high-quality fruit vinegar, including 3 European PGI products, namely, TBVM, BVM, and SV, and found that aging time had a very significant influence on their VVOCs. The VVOC profiles containing more than 100 VVOCs in 2 Italian PGI vinegar, namely TBVM “AFPfinato” (TBVM-AFP, aging>12 years) and TBVM “extravecchio” (TBVM-ST, aging>25 years) made by the cooked grape must be almost similar, but the contents of some VVOCs, such as (Z)-3-hexen-1-ol acetate, ethyl decanoate, decanoic acid, tetradecanoic acid, pentadecanoic acid, 9-hexa-decenoic acid, oleic acid, 5-hydroxymethylfurfural, 2, 3-dihydro-3, 5-dihydroxy−6-methyl-4H-pyran-4-one, and N, N-dimethyl-formamide, were significantly higher (*p* < 0.01) in TBVM-AFP than those in TBVM-ST, whereas neryl acetate, linalool, geraniol, isovalerone, and maltol were much higher (*p* < 0.01) in TBVM-ST than those in TBVM-AFP.

Liang et al. (2016) found that the VVOC profiles of SAV before and after aging were almost similar, but after aging, the levels of furan- and ester-like VVOCs from the Maillard reaction were greatly increased, leading that pungent smells were disappeared, and the higher aroma and flavor dilution factors of some VVOCs, including 3-(methylthio) propanal, vanillin, 2, 3-butanedione, tetramethylpyrazine, 3-methylbutanoic acid, γ-nonalactone, guaiacol, 3-(methylthio) propyl acetate, dimethyl trisulfide, phenylacetaldehyde, 2-ethyl-6-methylpyrazine, 2-acetylpyrazine, 2,3-dimethylpyrazine, furfural and 3-hydroxy-2-butanone, were appeared. Zhou et al. (2017) discovered that the concentrations of 2, 3-butanedione, 2-methylpropanal, sotolon, dimethyl trisulfide, 3-hydroxy-2-butanone, 2, 4, 5-trimethyloxazole and tetramethyl-pyrazine in ZAV, changed significantly during the aging process.

#### VVOCs From Other Processes

**Figure 6:**
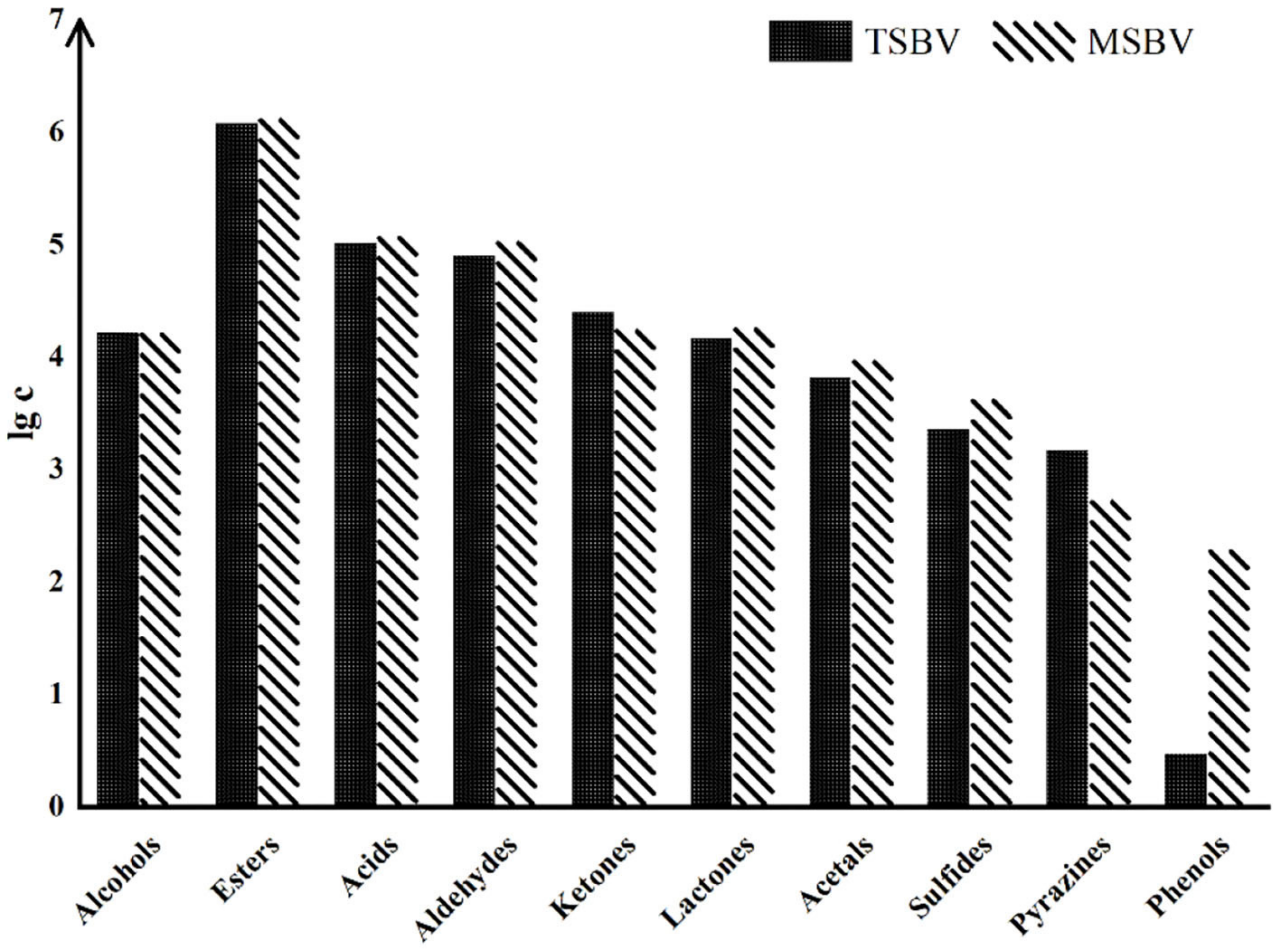
The logarithm of total concentration (μg/L) of the aroma-active chemical groups in both TSBV and MSBV samples (Cited and modified from Al-Dalai et al., 2020a).

Besides heating treatment and aging, other processes also have a great impact on VVOCs. For example, compared with the traditional brewing methods of some PGI vinegar products, their modern modified methods may cause an important influence on VVOCs, although their raw materials are exactly the same. Traditional vinegar product usually has a richer and more coordinated aroma and flavor than modern vinegar product. This may be mainly because in the production process of the modern vinegar product, in order to accelerate the production process, some microorganisms, such as yeasts and AAB, are usually inoculated and strengthened, leading that microbial diversities are greatly reduced during the process of modern vinegar. Moreover, the aging time of modern vinegar products is generally shorter than that of traditional vinegar products, which results in the fact that the aroma and flavor of modern vinegar products are usually far lower than those of traditional vinegar products, and its VVOCs diversities are also lower than those of traditional vinegar product. Zhao et al. (2018) evaluated the variations of VVOCs in TZAV and industrial Zhenjiang aromatic vinegar (IZAV) made with the same materials with different production methods. The TZAV production method is a slow and complicated fermentation process, which is controlled empirically to facilitate microbial growth and accumulation of VVOCs in vinegar, while the IZAV production method is a mechanical and fast production procedure. The authors found that with the extension of aging time (more than 3 years), TZAV and IZAV had proximate VVOC profiles, but total phenolic and flavonoid contents were higher in TZAV than those in IZAV, and rutin and *p*-coumaric acid were detected in TZAV but not in IZAV. Traditional Sichuan bran vinegar (TSBV) characterizes the starter growing on the upper layer of vinegar Pei (paste) in a wooden container. Contrarily in the modern Sichuan bran vinegar (MSBV) technology, microorganisms from the starter grow inside the vinegar Pei in an oblong cement pool and obtain oxygen for their growth by agitation to speed up the fermentation process. By comparing VVOCs from TSBV and MSBV, Al-Dalai et al. (2020a) found that TSBV samples have high concentrations of the VVOC groups—alcohols, ketones, and pyrazines, while the MSBV samples have high concentrations of the VVOCs groups—acids, esters, aldehydes, lactones, acetal, sulfides, and phenols (Figure 6). The results further discovered that 4-methylpentanoic acid, 3-(methylthio)-1-propanol, 1-phenyl-ethanone, 2-methoxyphenol, and benzothiazole were only identified in the TSBV samples, while 3-methylbutanal, 2-ethyl-1-hexanol, heptanoic acid, 4-ethyl-2-methoxyphenol and vanillin were only identified in the MSBV samples.

## Discussion and Perspective

Although many kinds of VVOCs in different kinds of vinegar, especially in the famous and traditional vinegar products have been investigated, which can outline the overall VVOCs characteristics, the VVOCs may be derived from their main raw materials, the relative microorganisms, and heating, aging, or other unique processes, moreover most of VVOCs dynamically change during the vinegar process. So it is a very difficult task to describe the formation processes of VVOCs. In addition, except VVOCs of some PGI vinegar products, such as SAV and ZAV from China, TBV, BV, and SV from Italy and Spain, respectively, which have been intensively investigated, the research on VVOCs from the other kinds of vinegar, such as CV, PV, BRV, JRV, KRV, and SBV, is inadequate. Therefore, the VVOCs data from CV, PV, BRV, JRV, KRV, and SBV (Annex Tables 1–3 and Figure 3), which we have collected in this review, might not be enough to reflect the aroma and flavor characteristics of these kinds of vinegar, and their VVOC profiles and forming processes need to further be modified according to more data achieved in future studies.

To better understand and study the VVOCs, in the future, at least the following aspects should be strengthened. (1) The isotope methods, such as stable isotopic fractionation-nuclear magnetic resonance and isotope ratio mass spectrometry, which have been used to differentiate the geographical origin of PGI vinegar products and the adulteration of kinds of vinegar (Cavdaroglu and Ozen, 2021), may also be applied to track the changes of important VVOCs during the vinegar brewing process. (2) The multi-omics technologies, including metagenomics, metaproteomics, and metabolomics, may be the future direction to elucidate how VVOCs are produced by microorganisms, and what are contributions of different microorganisms to VVOCs. (3) The multivariate statistical analysis methods, including principal component analysis, partial least squares discriminant analysis, and so on, have been applied to identify different kinds of vinegar, establish aroma fingerprints, and analyze characteristic aroma components of kinds of vinegar (Yin and Zhao, 2019; Zhang et al., 2019), therefore in the future the multivariate analysis of the VVOCs profile for discriminant features might be carried out to distinguish the different kinds of vinegar, especially PGI vinegar products.

## Author Contributions

XZ and CK contributed to the writing of the first draft. CK and HZ collected information and contributed to the tables. ZX contributed to the figures. CK, HZ, and ZX contributed in Supplementary Materials. FC give the overall idea to this manuscript as well as review and revise the manuscript. ZL and XZ give a guide as well as review the manuscript. All authors read, revise, and made final approval of the submitted version.

## Conflict of Interest

The authors declare that the research was conducted in the absence of any commercial or financial relationships that could be construed as a potential conflict of interest. The reviewer Z-HX declared a shared affiliation with one of the author ZL, to the handling editor.

## Publisher's Note

All claims expressed in this article are solely those of the authors and do not necessarily represent those of their affiliated organizations, or those of the publisher, the editors and the reviewers. Any product that may be evaluated in this article, or claim that may be made by its manufacturer, is not guaranteed or endorsed by the publisher.
