# Supplementary material for: Vinegar Volatile Organic Compounds: Analytical Methods, Constituents, and Formation Processes
Source: Front Microbiol. 2022 Jun 30;13:907883. doi: 10.3389/fmicb.2022.907883 (PMC9279916; doi:10.3389/fmicb.2022.907883)
Supplement: Supplementary file 1 [file Table_1.DOCX]

**Annex Table 1** **The main VOCs in fruit vinegars (160)**

| **Categories** | **VOCs** | **MF(MW)** | **Structural formula** | **Odor descriptor** | **ID** | **Vinegars** | **References** |
| --- | --- | --- | --- | --- | --- | --- | --- |
| **Acids (25)** | 2-Methyl butanoic acid | C_5_H_10_O_2_(102.13 ) |  | - | MS, GC, OD | BV, SV | Corsini et al., 2019; Roda et al., 2017; Pinu et al., 2016; Marrufo-Curtido et al., 2012; Acena et al.,2011; Cirlini et al., 2011; Truta et al., 2010; Callejón et al.,2008; Giordano et al., 2003. |
|  | 2-Methyl propionic acid | C_4_H_8_O_2_(88.11) |  | Rancid butter, cheese | MS, GC, OD | TBV, BV, SV |  |
|  | 3-Methyl butyric acid | C_5_H_10_O_2_(102.12) |  | Rancid, cheese | MS, GC, OD | TBV, BV, SV, PV |  |
|  | 4-Oxopentanoic acid | C_5_H_8_O_3_(116.12) |  | Caramel | MS, GC, OD | TBV, BV |  |
|  | 9-Hexadecenoic acid | C_16_H_30_O_2_(254.41) | 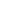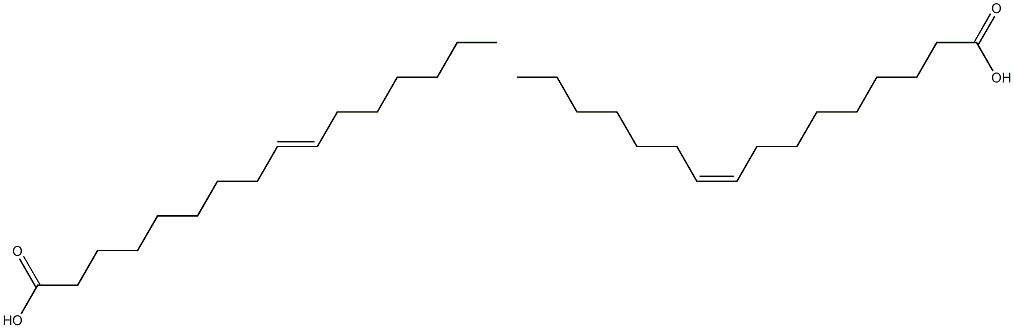 | - | MS, GC | TBV, SV |  |
|  | 9-Octadecenoic acid | C_18_H_34_O_2_ (282.46) |  | - | MS, GC | TBV |  |
|  | Acetic acid | C_2_H_4_O_2_(60.05) |  | Acidic, pungent, vinegar | MS, GC, OD | TBV, BV, SV, CV, PV |  |
|  | Benzoic acid | C_7_H_6_O_2_(122.12) |  | - | MS, GC | TBV, BV, SV |  |
|  | Butanoic acid | C_4_H_8_O_2_(88.11) |  | Rancid, butter | MS, GC, OD | TBV, BV, SV |  |
|  | (E)-But-2-enoic acid | [C_4_H_6_O_2_ (86.09)](https://pubchem.ncbi.nlm.nih.gov/#query=C4H6O2) |  | Milk product, roasted, burnt | MS, GC, OD | TBV, BV |  |
|  | Decanoic acid | C_10_H_20_O_2_(172.26) |  | Chamomile tea, sweet | MS, GC, OD | TBV, BV, SV |  |
|  | Formic acid | CH_2_O_2_(46.03) |  | - | MS, GC | TBV |  |
|  | Furoic acid | C_5_H_4_O_3_(112.08) |  | Fatty, musty, waxy | MS, GC, OD | TBV, BV |  |
|  | Hexanoic acid | C_6_H_12_O_2_(116.16) |  | Cheesy, goaty, sweat | MS, GC, OD | TBV, BV, SV, PV |  |
|  | Hexadecanoic acid | C_16_H_32_O_2_ (256.43) |  | - | MS, GC | TBV, BV, SV |  |
|  | Heptanoic acid | C_7_H_14_O_2_(130.18) |  | Cheese | MS, GC, OD | BV, SV |  |
|  | Octanoic acid | C_8_H_16_O_2_(144.21) |  | Fatty, rancid, vegetable | MS, GC, OD | TBV, BV, SV, PV |  |
|  | Octadecanoic acid | C_18_H_36_O_2_(284.48) |  | - | MS, GC | TBV |  |
|  | Oleic acid | C_18_H_34_O_2_ (282.46) |  | - | MS, GC | TBV |  |
|  | Pentadecanoic acid | C_15_H_30_O_2_(242.40) |  | - | MS, GC | TBV, BV, SV |  |
|  | Pentanoic acid | C_5_H_10_O_2_ (102.13) |  | - | MS, GC | BV, SV |  |
|  | Phenylacetic acid | C_8_H_8_O_2_(136.15) |  | Sweet, animal, honey-like | MS, GC, OD | TBV, BV |  |
|  | Propanoic acid | C_3_H_6_O_2_(74.08) |  | Pungent, rancid | MS, GC, OD | TBV, BV, PV, SV |  |
|  | Nonanoic acid | C_9_H_18_O_2_(158.24) |  | - | MS, GC | TBV, BV, SV |  |
|  | Tetradecanoic acid | C_14_H_28_O_2_( 228.37) |  | - | MS, GC | TBV, BV, SV |  |
| **Alcohols (22)** | 1-Hexanol | C_6_H_14_O（102.17） |  | Floral | MS, GC, OD | SV, CV, PV |  |
|  | 1-Pentanol | C_5_H_12_O（88.15） |  | - | MS, GC | PV |  |
|  | 1-Propanol | C_3_H_8_O（60.09） |  | - | MS, GC | BV, SV |  |
|  | 2, 3-Butanediol | C_4_H_10_O_2_（90.12） |  | Fruity, creamy, buttery | MS, GC, OD | TBV, BV, SV,PV |  |
|  | 2-Butoxy-ethanol | [C_6_H_14_O_2_（118.17）](https://www.chembk.com/cn/search/C6H14O2) |  | - | MS, GC | PV |  |
|  | 2-Ethyl-1-hexanol | C_8_H_18_O（130.23） |  | - | MS, GC | PV |  |
|  | 2-Furanmethanol | C_5_H_6_O_2_（98.10） |  | Burned, burned hair | MS, GC, OD | BV, SV |  |
|  | 2-Hexanol | C_6_H_14_O（102.17） |  | Chemical, winey, cauliflower | MS, GC, OD | TBV, BV |  |
|  | 2-Methyl-1-propanol | C_4_H_10_O（74） |  | Alcohol, wine | MS, GC, OD | TBV, BV, SV PV |  |
|  | 2-Methyl-1-butanol | C_5_H_12_O（88.15） |  | - | MS, GC | TBV, BV, SV,CV |  |
|  | 3-Ethoxy-1-propanol | C_7_H_18_O_3_（150.20） |  | - | MS, GC | TBV |  |
|  | 3-Methyl-1-butanol | C_5_H_12_O(88.15) |  | Alcohol | MS, GC, OD | TBV, BV, SV, CV, PV |  |
|  | 3-Methylthio-propanol | C_4_H_10_OS(106.19) |  | - | MS, GC | BV,PV |  |
|  | Benzyl alcohol | C_7_H_8_O(108.14) |  | Slightly pungent, fruity | MS, GC, OD | TBV, BV, SV, |  |
|  | Borneol | C_10_H_18_O(154.25) | 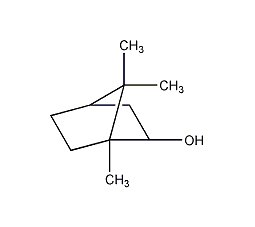 | - | MS, GC | TBV, BV, SV |  |
|  | Cis-3-hexen-1-ol | C_6_H_12_O(100.16) |  | - | MS, GC | BV,SV |  |
|  | Ethanol | C_2_H_6_O(46.07) |  | Alcohol | MS, GC, OD | BV, SV |  |
|  | Fenchyl alcohol | C_10_H_18_O(154) |  | - | MS, GC | TBV, BV, SV |  |
|  | Methanol | CH_4_O(32.04) |  | - | MS, GC | BV, SV |  |
|  | Methionol | C_4_H_10_OS(106.19) |  | Boiled vegetable or potatoes | MS, GC, OD | BV, SV |  |
|  | Octanol | C_8_H_18_O(130.22) |  | - | MS, GC | PV |  |
|  | Phenylethyl alcohol | C_8_H_10_O（122.17） |  | Mild, warm, rose, honey-like | MS, GC, OD | TBV, BV, SV, PV |  |
| **Aldehydes (17)** | 2-Hexenal | C_6_H_10_O(98.15) | 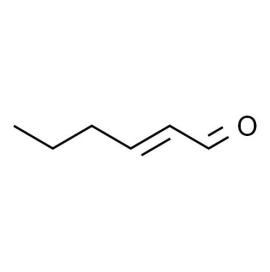 | - | MS, GC | CV |  |
|  | 3-Methyl benzaldehyde | C_8_H_8_O(120.15) |  | - | MS, GC | PV |  |
|  | 3-Methyl butanal | C_5_H_10_O(86.13) |  | - | MS, GC | BV, SV |  |
|  | 5-Acetoxymethyl-2-furaldehyde | C_8_H_8_O_4_(168.15) |  | Sweet | MS, GC, OD | TBV, BV，SV |  |
|  | 5-Ethoxymethylfurfural | C_8_H_10_O_3_(154.17) |  | - | MS, GC | TBV, BV, SV |  |
|  | 5-Hydroxymethylfurfural | C_6_H_6_O_3_(126.11) |  | - | MS, GC | TBV, BV, SV |  |
|  | 5-Methyl furfural | C_6_H_6_O_2_(110.11) |  | - | MS, GC | TBV, BV, SV |  |
|  | Acetaldehyde | C_2_H_4_O(44.05) |  | - | MS, GC | BV,SV |  |
|  | Benzaldehyde | C_7_H_6_O(106.12) |  | Almond | MS, GC, OD | TBV,BV,SV,CV |  |
|  | Decanal | C_10_H_10_O(156.27) |  | - | MS, GC | BV |  |
|  | Furfural | C_5_H_4_O_2_(96.09) |  | Alcoholic, sweet | MS, GC, OD | TBV, BV, SV |  |
|  | Hexanal | C_6_H_12_O(100.16) |  | - | MS, GC | BV,SV,CV |  |
|  | Methional | C_4_H_8_OS(104.17) |  | Boiled potato | MS, GC, OD | SV |  |
|  | Nonanal | C_9_H_18_O(142.24) |  | - | MS, GC | BV,SV |  |
|  | Octanal | C_8_H_16_O(128.22) |  | - | MS, GC | TBV, BV, SV |  |
|  | Phenylacetaldehyde | C_8_H_8_O(120.15) |  | - | MS, GC | BV |  |
|  | Vanillin | C_8_H_8_O3(152.15) |  | Sweet, creamy, vanilla | MS, GC, OD | TBV, BV, SV |  |
| **Esters (53)** | 1, 2-Propanediol diacetate | C_7_H_12_O_4_(160.17) |  | - | MS, GC | TBV, BV, SV |  |
|  | 1, 3-Propanediol diacetate | C_7_H_12_O_4_(160.17) |  | - | MS, GC | BV |  |
|  | 2-Methyl-propyl acetate | C_6_H_12_O_2_(116.16) |  | Fruity | MS, GC, OD | TBV, BV, SV, CV,PV |  |
|  | 2-Phenylethyl acetate | C_10_H_12_O_2_(164.20) |  | Sweet, rosy-fruity, honey-like | MS, GC, OD | TBV, BV, SV, PV |  |
|  | 2-Methyl butanol acetate | C_7_H_14_O_2_(130.19) |  | - | MS, GC | BV, CV |  |
|  | 2,3-Butanediol diacetate | C_8_H_14_O_4_(174.20) |  | Toasted maize, fried chicken | MS, GC, OD | TBV, BV, SV |  |
|  | 3-Methylbutyl acetate | [C_7_H_14_O_2_](https://www.chembk.com/cn/search/C7H14O2)(130.185) |  | banana, mulberry, strawberry | MS,GC | TBV,BV,SV,CV,PV |  |
|  | 3-Methylbutyl pentanoate | C_10_H_20_O_2_(172.27) |  | - | MS, GC | CV |  |
|  | 4-Methyl-2-pentyl acetate | C_8_H_16_O_2_(144.21) |  | - | MS, GC | TBV,BV,SV |  |
|  | Amyl acetate | C_7_H_14_O2(130.19) |  | Banana | MS, GC, OD | SV |  |
|  | Benzyl acetate | C_9_H10O_2_(150.18) |  | Sweet, fruity, floral | MS, GC, OD | TBV, BV, SV |  |
|  | Bornyl acetate | C_12_H_20_O_2_(196.29) |  | - | MS, GC | TBV, SV |  |
|  | Butyl acetate | C_6_H_12_O_2_(116.16) |  | Cherry, strawberry | MS, GC, OD | BV , SV |  |
|  | Diethyl succinate | C_8_H_14_O_4_(174.20) |  | Fruit, banana | MS, GC, OD | TBV, BV, SV |  |
|  | Dihydroxymethyl jasmonate | C_14_H_21_O_5_**^-^**(269.32) |  | - | MS, GC | TBV, BV, SV |  |
|  | Ethyl 2-methylbutyrate | C_7_H_14_O_2_(130.19) |  | - | MS, GC | SV, CV |  |
|  | Ethyl 2-methylpropanoate | C_6_H_12_O_2_(116.16) |  | Strawberry | MS, GC, OD | BV,SV, CV |  |
|  | Ethyl 3-hydroxyhexanoate | C_8_H_16_O_3_(160.21) |  | - | MS, GC | PV |  |
|  | Ethyl 3-hydroxybutanoate | C_6_H_12_O_3_(132.16) |  | - | MS, GC | SV |  |
|  | Ethyl 3-methylbutyrate | C_7_H_14_O_2_(130.19) |  | Strawberry | MS, GC, OD | TBV, BV, SV, CV |  |
|  | Ethyl 3-methylthio-propanoate | C_6_H_12_OS(132.22) |  | - | MS, GC | PV |  |
|  | Ethyl acetate | C_4_H_8_O_2_(88.11) |  | Soap, grass | MS, GC, OD | TBV, BV, SV, CV, PV |  |
|  | Ethyl acetoacetate | C_6_H_10_O_3_(130.14) |  | - | MS, GC | BV |  |
|  | Ethyl benzoate | C_9_H_10_O_2_(150.18) |  | Sweet | MS, GC, OD | SV, PV |  |
|  | Ethyl butanoate | C_6_H_12_O_2_(116.16) |  | Fruity, sweet, strawberry | MS, GC, OD | TBV, BV, SV, PV |  |
|  | Ethyl decanoate | C_12_H_24_O_2_(200.32) |  | - | MS, GC | TBV,BV, SV, PV |  |
|  | Ethyl furoate | C_7_H_8_O_3_(140.14) |  | - | MS, GC | BV , SV |  |
|  | Ethyl heptanoate | C_9_H_18_O_2_(158.24) |  | - | MS, GC | SV, PV |  |
|  | Ethyl hexanoate | C_8_H_16_O_2_(144.21) |  | Banana, fruit, mulberry | MS, GC, OD | TBV, BV, SV,CV,PV |  |
|  | Ethyl valerate | C_7_H_14_O_2_(130.19) |  | - | MS, GC | BV, SV |  |
|  | Ethyl lactate | C_5_H_10_O_3_(118.13) |  | Sweet, fruity, acidic | MS, GC, OD | TBV, BV, SV |  |
|  | Ethyl levulinate | C_7_H_12_O_3_(144.17) |  | - | MS, GC | TBV,BV, SV |  |
|  | Ethyl nonanoate | C_11_H_22_O_2_(186.30) |  | Aspirin, mulberry, cherry | MS, GC, OD | SV |  |
|  | Ethyl octanoate | C_10_H_20_O_2_(172.27) |  | Strawberry, banana | MS, GC, OD | TBV, BV, SV, PV |  |
|  | Ethyl phenylacetate | C_10_H_12_O_2_(164.20) |  | Floral, honey, sweet | MS, GC, OD | TBV , BV, SV, PV |  |
|  | Ethyl propanoate | C_5_H_10_O_2_(102.13) |  | - | MS, GC | BV, SV, CV |  |
|  | Ethyl salicylate | C_9_H_10_O_3_(166.18) |  | Urine | MS, GC, OD | SV |  |
|  | Ethyl vanillate | C_10_H_12_O_4_(196.20) |  | - | MS, GC | TBV, BV, SV |  |
|  | Geranyl acetate | C_12_H_20_O_2_(196.29) |  | - | MS, GC | TBV, BV, SV |  |
|  | Hexyl acetate | C_8_H_16_O_2_(144.21) |  | Mulberry, banana | MS, GC, OD | TBV, BV, SV，CV |  |
|  | Isoamyl acetate | C_7_H_14_O_2_(130.19) |  | Banana, fruity | MS, GC, OD | TBV , BV,SV,CV,PV |  |
|  | Isobutyl isothiocyanate | C_5_H_9_NS(115.19) |  | Fruity | MS, GC, OD | TBV, BV, SV |  |
|  | Methyl 3-hydroxy-2-methyl-butanoate | C_6_H_12_O_3_(132.16) |  | - | MS, GC | PV |  |
|  | Methyl 3-methylthio-propanoate | C_5_H_10_OS(118.19) |  | - | MS, GC | PV |  |
|  | Methyl acetate | C_3_H_6_O_2_(74.08) |  |  | MS, GC | BV , SV |  |
|  | Methyl furan-2-carboxylate | C_6_H_6_O_3_(126.11) |  | - | MS, GC | BV |  |
|  | Methyl hexanoate | C_7_H_14_O_2_(130.19) |  | - | MS, GC | TBV, SV |  |
|  | methyl salicylate | C_8_H_8_O_3_(152.15) |  | - | MS.GC | TBV,BV,SV |  |
|  | Neryl acetate | C_12_H_20_O_2_(196.29) |  | - | MS, GC | TBV, BV, SV |  |
|  | Propyl acetate | C_5_H_10_O_2_(102.13) |  | - | MS, GC | BV ,SV |  |
|  | Vinyl acetate | C_4_H_6_O_2_(86.09) |  | - | MS, GC | CV |  |
|  | (E)-2-Hexen-1-ol acetate | C_8_H_14_O_2_(142.20) |  | - | MS, GC | TBV, SV |  |
|  | (Z)-3-Hexen-1-ol acetate | C_8_H_14_O_2_(142.20) |  | - | MS, GC | TBV, BV, SV |  |
| **Ketones (20)** | 1-(2 ,3, 6-Trimethylphenyl)-3-buten-2-one | C_13_H_16_O(188.27) |  | - | MS, GC | TBV, BV, SV |  |
|  | 1-(5-methyl-2-furyl)-1-propanone | C_8_H_10_O_2_(138.17) |  | - | MS, GC | TBV,BV,SV |  |
|  | 2,3-Butanedione | C_4_H_6_O_2_(86.09) |  | Sweet, toasted | MS, GC, OD | TBV, BV, SV |  |
|  | 2-Hydroxy-3-methyl-cyclopent-2-en-1-one | C_6_H_8_O_2_(112.13) |  | Sweet, caramel, maple | MS, GC, OD | TBV, BV |  |
|  | 2-Methyloxolan-3-one | C_5_H_8_O_2_(100.12) |  | Sugar, coffee | MS, GC, OD | TBV, BV |  |
|  | 2-Methyl-3-hydroxy-4-pyrone | C_6_H_6_O_3_(126.11) |  | Butter | MS, GC, OD | SV |  |
|  | 2-Nonanone | C_9_H_18_O(142.24) |  | - | MS, GC | PV |  |
|  | 3, 5-Dihydroxy-2-methyl-(4H)-pyran-4-one | C_6_H_6_O_4_(142.11) |  | Toasted, caramel | MS, GC, OD | TBV, BV |  |
|  | 3-Hydroxy-2-butanone | C_4_H_8_O_2_(88.11) |  | Sweet, vanilla | MS, GC, OD | TBV, BV, SV,PV |  |
|  | 3-Hydroxy-2-pentanone | C_5_H_10_O_2_(102.13) |  | Buttery | MS, GC, OD | SV |  |
|  | 4-Methyl heptanone | C_8_H_16_O(128.22) |  | - | MS, GC | PV |  |
|  | 5, 6-Dihydro-4-methyl-(2H)-pyran-2-one | C_6_H_8_O_2_(112.13) |  | Sweet | MS, GC, OD | TBV, BV |  |
|  | 5-Methyl-3-hexanone | C_7_H_14_O(114.19) |  | - | MS, GC | TBV, BV, SV |  |
|  | 6-Methyl 5-hepten-2-one | C_8_H_14_O(126.20) |  | - | MS, GC | BV, CV |  |
|  | Acetophenone | C_8_H_8_O(120.15) |  | - | MS, GC | SV |  |
|  | Benzophenone | C_13_H_10_O(182.22) |  | - | MS, GC | TBV,BV,SV |  |
|  | Camphor | C_10_H_16_O(152.24) |  | - | MS, GC | CV |  |
|  | Isovalerone | C_9_H_18_O(142.24) |  | - | MS, GC | TBV,BV,SV |  |
|  | Neryl acetone | C_13_H_22_O(194.32) |  | - | MS, GC | BV |  |
|  | β-Damascenone | C_13_H_18_O(190.29) |  | Stewed apples, apple juice | MS, GC, OD | SV |  |
| **Lactones (10)** | α-Methyl-γ-crotonolactone | C_5_H_6_O_2_(98.10) |  | - | MS, GC | TBV, BV, SV |  |
|  | *Trans-*β-methyl-γ-octalactone | C_9_H_16_O_2_(156.23) |  | - | MS, GC | BV, SV |  |
|  | Cis-β-methyl-γ-octalactone | C_9_H_16_O_2_(156.23) |  | - | MS, GC | BV, SV |  |
|  | γ-Butyrolactone | C_4_H_6_O_2_(86.09) |  | - | MS, GC | TBV, BV, SV |  |
|  | γ-Decalactone | C_10_H_18_O_2_(170.25) |  | Coconut, sweet | MS, GC, OD | SV |  |
|  | γ-Heptalactone | C_7_H_12_O_2_(128.17) |  | - | MS, GC | TBV, BV, SV |  |
|  | δ-2-Decenolactone | C_10_H_16_O_2_(168.24) |  | - | MS, GC | TBV, BV, SV |  |
|  | δ-Laurolactone | C_12_H_22_O_2_(198.31) |  | - | MS, GC | TBV, BV, SV |  |
|  | δ-Decalactone | C_10_H_18_O_2_(170.25) |  | - | MS, GC | TBV, BV, SV |  |
|  | Sotolon (Sugar lactone) | C_6_H_8_O_3_(128.13) |  | Curry, liquorice, syrupy | MS, GC, OD | SV |  |
| **Phenols (11)** | 2,4-Ditertbutyl phenol | C_14_H_22_O(206.33) |  | - | MS, GC | TBV, BV, SV |  |
|  | 4-Acetyl-2-methylphenol | C_9_H_10_O_2_(150.18) |  | - | MS, GC | TBV, BV, SV |  |
|  | 4-Ethyl phenol | C_8_H_10_O(122.17) |  | Metallic | MS, GC, OD | TBV, BV, SV, PV |  |
|  | 4-Ethylguaiacol | C_9_H_12_O_2_(152.19) |  | Flower (daisy),chamomile tea | MS, GC, OD | TBV, BV, SV |  |
|  | 4-Vinyl guaiacol | C_9_H_10_O_2_(150.18) |  | - | MS, GC | SV,PV |  |
|  | 4-Vinyl phenol | C_8_H_8_O(120.15) |  | - | MS, GC | PV |  |
|  | 4-Ethyl resorcinol | C_8_H_10_O_2_(138.17) |  | - | MS, GC | TBV,BV,SV |  |
|  | Eugenol | C_10_H_12_O_2_(164.20) |  | Clove, cinnamon | MS, GC, OD | TBV, BV, SV, PV |  |
|  | Guaiacol | C_7_H_8_O_2_(124.14) |  | - | MS, GC | BV,SV |  |
|  | Maltol | C_6_H_6_O_3_(126.11) |  | Toasted maize | MS, GC, OD | TBV,BV |  |
|  | Phenol | C_6_H_6_O(94.11) |  | - | MS, GC | TBV, BV, SV, PV |  |
| **Pyrazine (1)** | 2, 5-Dimethylpyrazine | C_6_H_8_N_2_(108.14) |  | - | MS, GC | PV |  |
| **Furan (1)** | 2-Acetylfuran | C_6_H_6_O_2_(110.11) |  | Sweet, balsamic, almond | MS, GC, OD | TBV, BV, SV |  |

VOCs: Volatile organic compounds; MF:Molecular formula; MW: Molecular weight; ID: Identification; MS: Mass spectrometry; GC: Gas chromatography; OD: Odor description at the olfactory detection port; TBV: Traditional balsamic vinegar; BV: Balsamic vinegar; SV: Sherry vinegar; CV: Cider vinegar; PV: Pineapple vinegar.

**Annex Table 2 The main VOCs in cereal vinegars (124)**

| **Categories** | **VOCs** | **MF(MW)** | **Structural formula** | **Odor descriptor** | **ID** | **Vinegars** | **References** |
| --- | --- | --- | --- | --- | --- | --- | --- |
| **Acids (15)** | 2-Furoic acid | C_5_H_4_O_3_(112.08) |  | Fatty, acid | MS, GC, OD | ZAV | Al-Dalai et al., 2020; Zhou et al., 2020; Zhang et al., 2019; Al-Dalai et al., 2019a, 2019b; Zhao et al., 2018; Zhu et al., 2018; Chung et al., 2017; Wang et al., 2017; Zhou et al., 2017; Liang et al., 2016; Zhu et al., 2016; Xiao et al., 2011. |
|  | 2-Methylbutanoic acid | C_5_H_10_O_2_(102.13) |  | Smelly, stinky,acid | MS, GC, OD | SBV |  |
|  | 2-Methylpropionic acid | C_7_H_14_O_2_(130.18) |  | Sour, acid | MS, GC, OD | ZAV, SBV, KRV, JRV |  |
|  | 3-Methylbutyric acid | C_4_H_8_O_2_(88.11) |  | Stinky, acid, sour | MS, GC, OD | SAV, ZAV, SBV, KRV, JRV |  |
|  | 3-(Methylthio) propionic acid | C₄H₈O₂S(120.17) |  | Burnt, roasted | MS, GC, OD | ZAV |  |
|  | 4-Methylpentanoic acid | C_6_H_12_O_2_(116.16) |  | Stinky | MS, GC, OD | SBV |  |
|  | Acetic acid | C_2_H_4_O_2_(60.05) |  | Acidic, vinegar, pungent | MS, GC, OD | SAV, ZAV, SBV, BRV, KRV, JRV |  |
|  | Butanoic acid | C_4_H_8_O_2_(88.11) |  | Rancid, butter | MS, GC, OD | SAV, ZAV, KRV |  |
|  | Heptanoic acid | C_7_H_14_O_2_(130.18) |  | Sour, smelly | MS, GC, OD | SBV |  |
|  | Hexanoic acid | C_6_H_12_O_2_(116.16) |  | Acid, mold, sour | MS, GC, OD | SAV, ZAV, SBV , BRV, KRV, JRV |  |
|  | Nonanoic acid | C_9_H_18_O_2_(158.24) |  | - | MS, GC | KRV, JRV |  |
|  | Pentanoic acid | C_5_H_10_O_2_(102.13) |  | Sour, cheesy | MS, GC, OD | SAV, ZAV |  |
|  | Propanoic acid | C_3_H_6_O_2_(74.078) |  | Pungent, rancid | MS, GC, OD | SAV, ZAV, KRV, JRV |  |
|  | Phenylacetic acid | C_8_H_8_O_2_(136.15) |  | - | MS, GC | JRV |  |
|  | Octanoic acid | C_8_H_16_O_2_(144.2) |  | Rancid | MS, GC, OD | ZAV, SBV, BRV KRV, JRV |  |
| **Alcohols (14)** | 1-Hexanol | C_6_H_14_O（102.2） |  | Plant, green | MS, GC, OD | ZAV, BRV |  |
|  | 1-Propanol | C_3_H_8_O（60.1） |  | Alcohol | MS, GC, OD | SAV |  |
|  | 2, 3-Butanediol | C_4_H_10_O_2_（90.1） |  | Fruity, creamy, buttery | MS, GC, OD | SAV, ZAV |  |
|  | 2-Butanol | C_4_H_10_O（74.1） |  | - | MS, GC | SAV，ZAV |  |
|  | 2-Ethyl-1-hexanol | [C_6_H_14_O_2_(118.2）](https://www.chembk.com/cn/search/C6H14O2) |  | Fruity | MS, GC, OD | SAV, ZAV, SBV |  |
|  | 2-Ethyl-2-hexanol | C_8_H_18_O（130.2） |  | - | MS, GC | ZAV |  |
|  | 2-Furanmethanol | C_5_H_6_O_2_(98.1) |  | Caramel,coffee,burned,burned hair | MS, GC, OD | SAV, ZAV, SBV |  |
|  | 2-Methyl-1-butanol | C_5_H_12_O(88.15) |  | - | E-nose | KRV, JRV |  |
|  | 2-Methyl-1-propanol | C_4_H_10_O（74） |  | Nail polish | MS, GC, OD | SAV, ZAV, BRV, KRV,JRV |  |
|  | 3-Methyl-1-butanol | C_5_H_12_O(88.1) |  | Alcohol | MS, GC, OD | SAV, ZAV,BRV,KRV,JRV |  |
|  | 3-(Methylthio)-1-propanol | C_4_H_10_OS(106.2) |  | Fruity, Cooked vegetable | MS, GC, OD | SAV, ZAV, SBV |  |
|  | Benzyl alcohol | C_7_H_8_O(108.1) |  | Floral, rose, honey | MS, GC, OD | SAV, ZAV, SBV |  |
|  | Ethanol | C_2_H_6_O(46.1) |  | Alcohol | MS, GC, OD | SAV, ZAV, BRV,KRV,JRV |  |
|  | Phenylethyl alcohol | C_8_H_10_O(122.17) |  | Floral, rose, honey | MS, GC, OD | SAV, ZAV, SBV, BRV, KRV, JRV |  |
| **Aldehydes (29)** | (E)-3-(Furan-2-yl)-2-methylprop-2-enal | C_8_H_8_O_2_(136.15) |  | Herbal | MS, GC, OD | SBV |  |
|  | 1H-pyrrole-2-carboxaldehyde | C_5_H_5_NO(95.1) |  | Smelly, coffee | MS, GC, OD | SBV |  |
|  | 2-Methylbutanal | C_5_H_10_O(86.13) |  | - | MS, GC | SAV, ZAV |  |
|  | 2-Methylpropanal | C_4_H_8_O(72.1) |  | - | MS, GC | SAV, ZAV |  |
|  | 2-Methylbenzaldehyde | C_8_H_8_O(120.15) |  | - | MS, GC | KRV, JRV |  |
|  | 2-Phenyl-2-butenal | C_10_H_10_O(146.19) | 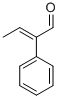 | Sweet, floral, coconut | MS, GC, OD | ZAV, SBV |  |
|  | 2-Phenyl-3-(2-furyl)-propenal | C_13_H_10_O_2_(198.22) |  | Spicy | MS, GC, OD | SBV |  |
|  | 2-Phenylacetaldehyde | C_8_H_8_O(120.15) |  | Green,honey, sweet, floral | MS, GC, OD | SAV, ZAV, SBV |  |
|  | 3-Methylbutanal | C_5_H_10_O(86.13) |  | Acid, sour, sweet | MS, GC, OD | SAV, ZAV, SBV, KRV |  |
|  | 3-(Methylthio)-propanal | C_4_H_8_OS(104.17) |  | Onion meat mashed potatoes | MS | ZAV |  |
|  | 4-Methylbenzaldehyde | C_8_H_8_O(120.15) |  | - | MS, GC | KRV, JRV |  |
|  | 4-Methoxybenzaldehyde | C_8_H_8_O_2_(136.15) |  | - | MS, GC | SAV, ZAV |  |
|  | 5-Hydroxymethylfurfural | C_6_H_6_O_3_(126.11) |  | Buttery,fatty, bittery | MS, GC, OD | ZAV, SBV |  |
|  | 5-Methyl-2-phenyl-2-hexenal | C_13_H_16_O(188.27) |  | Bitter | MS, GC, OD | SBV |  |
|  | 5-Methylfurfural | C_6_H_6_O2(110.11) |  | Caramel,spicy | MS, GC, OD | SAV, ZAV, SBV |  |
|  | Acetaldehyde | C_2_H_4_O(44.05) |  | - | MS, GC | SAV, ZAV, KRV, JRV |  |
|  | Benzaldehyde | C_7_H_6_O(106.12) |  | Almond | MS, GC, OD | SAV, ZAV, KRV, JRV |  |
|  | Cinnamaldehyde | C_9_H_8_O(132.16) |  | Sweet, burnt sugar | MS, GC, OD | SAV, ZAV |  |
|  | Decanal | C_10_H_10_O(156.27) |  | Sweet, floral | MS, GC, OD | ZAV |  |
|  | Dodecanal | C_12_H_24_O(184.32) |  | Mud, stinky | MS, GC, OD | KRV, JRV, |  |
|  | Furfural | C_5_H_4_O_2_(96.09) |  | Burnt sugar, bitter,almond | MS, GC, OD | SAV, ZAV, SBV, BRV, KRV, JRV |  |
|  | Hexanal | C_6_H_12_O(100.16) |  | Grassy, green | MS, GC, OD | SAV, ZAV |  |
|  | Methional | C_4_H_8_OS(104.17) |  | Cooked vegetable | MS, GC, OD | SAV |  |
|  | Nonanal | C_9_H_18_O(142.24) |  | Fresh | MS, GC, OD | ZAV |  |
|  | Octanal | C_8_H_16_O(128.22) |  | Aldehydic,waxy,citrus,fatty | MS | ZAV |  |
|  | Propionaldehyde | C_3_H_6_O(58.05) |  | - | E-nose | KRV, JRV |  |
|  | Safranal | C_10_H_14_O(150.22) |  | Herbal | MS, GC, OD | ZAV |  |
|  | Vanillin | C_8_H_8_O_3_(152.15) |  | Sweet, vanilla | MS, GC, OD | SAV, ZAV, SBV |  |
|  | (Z)-2-Nonenal | C_9_H_16_O(140.23) |  | Green, fatty | MS, GC, OD | SAV |  |
| **Esters (32)** | 2-Butyl acetate | C_6_H_12_O_2_(116.16) |  | - | MS, GC | SAV, ZAV |  |
|  | 2-Furanmethanol-acetate | C_7_H_8_O_3_(140.14) |  | Sweety, butter Banana, sweet | MS, GC, OD | ZAV, SBV |  |
|  | 2-Methylpropyl acetate | C_7_H_14_O_2_(130.19) |  | Banana, fruity | MS, GC, OD | SAV, ZAV, BRV, KRV, JRV |  |
|  | 3-(Acetyloxy) butan-2-yl acetate | C_8_H_14_O_4_(174.20) |  | Smoke | MS, GC, OD | ZAV |  |
|  | 3-Methylbutyl acetate | C_6_H_12_O_2_(116.16) |  | Butter | MS, GC, OD | SAV, ZAV, BRV, KRV, JRV |  |
|  | 3-(Methylthio)propyl acetate | C_6_H_12_O_2_S(148.22) |  | Herbal | MS, GC, OD | SAV, ZAV |  |
|  | Butyl benzoate | C_11_H_14_O_2_(178.23) |  | Creamy, buttery | MS, GC, OD | ZAV |  |
|  | Diethyl succinate | C_8_H_14_O_4_(174.20) |  | Fruit | MS, GC, OD | SAV, ZAV, BRV, KRV, JRV |  |
|  | Ethyl 2-hydroxy-4-methylpentanoate | C_8_H_16_O_3_(160.21) |  | Creamy, buttery | MS, GC, OD | ZAV |  |
|  | Ethyl 2-methylbutyrate | C_7_H_14_O_2_(130.19) |  | - | MS, GC, OD | JRV |  |
|  | Ethyl 3-methylbutyrate | C_7_H_14_O_2_(130.19) |  | - | MS, GC | ZAV |  |
|  | Ethyl 4-acetoxybutanoate | C_8_H_14_O_4_(174.20) |  | - | MS, GC | ZAV |  |
|  | Ethyl acetate | C_4_H_8_O_2_(88.11) |  | Cheese, feet | MS, GC, OD | SAV, ZAV, BRV, KRV, JRV |  |
|  | Ethyl benzoate | C_9_H_10_O_2_(150.18) |  | - | MS, GC | SAV, ZAV, BRV |  |
|  | Ethyl decanoate | C_12_H_24_O_2_(200.32) |  | Sweet, fruity | MS, GC, OD | ZAV |  |
|  | Ethyl hexanoate | C_8_H_16_O_2_(144.21) |  | Fruity, strawberry | MS, GC, OD | BRV, ZAV |  |
|  | Ethyl lactate | C_5_H_10_O_3_(118.13) |  | Sweet, fruity | MS, GC, OD | SAV, ZAV, SBV, KRV |  |
|  | Ethyl octanoate | C_10_H_20_O_2_(172.27) |  | Banana, fruity | MS, GC, OD | SAV, ZAV |  |
|  | Ethyl phenylacetate | C_10_H_12_O_2_(164.20) |  | Fruity, buttery | MS, GC, OD | SAV, ZAV, BRV, KRV |  |
|  | Ethyl propanoate | C_5_H_10_O_2_(102.13) |  | Strawberry, banana | MS, GC, OD,  E-nose | SAV, KRV, JRV |  |
|  | Hexyl acetate | C_8_H_16_O_2_(144.21) |  | Fruity, sweet | MS, GC, OD | ZAV |  |
|  | Methyl 2-methylbutyrate | C_6_H_12_O_2_(116.16) |  | Fruity, flowery | MS, GC, OD | ZAV |  |
|  | [Methyl 2-methylpropanoate](https://www.chembk.com/en/chem/methyl%202-methylpropanoate) | C_5_H_10_O_2_(102.13) |  | - | MS, GC, OD, E-nose | KRV |  |
|  | Methyl acetate | C_3_H_6_O_2_(74.08) |  | - | MS, GC, E-nose | SAV, ZAV, KRV, JRV |  |
|  | Methyl butyrate | C_5_H_10_O_2_(102.13) |  | Strawberry, fruity | MS, GC, OD | ZAV |  |
|  | Methyl cinnamate | C_10_H_10_O_2_(162.19) |  | - | MS, GC | SAV, ZAV |  |
|  | Methyl furan-2-carboxylate | C_6_H_6_O_3_(126.11) |  | Plastic | MS, GC, OD | ZAV |  |
|  | Monomethyl succinate | C_14_H_24_O_4_(256.34) |  | - | MS, GC | ZAV |  |
|  | N-pentyl acetate | C_7_H_14_O_2_(130.19) |  | - | MS, GC | ZAV |  |
|  | Phenylethyl acetate | C_10_H_12_O_2_(164.2) |  | Fruity | MS, GC, OD | SAV, ZAV, SBV, BRV, KRV, JRV |  |
|  | Propyl acetate | C_5_H_10_O_2_(102.13) |  | - | MS, GC | SAV, ZAV |  |
|  | Vinyl acetate | C_4_H_6_O_2_(86.09) |  | Acid, mold, sour | MS, GC, OD | KRV, JRV |  |
| **Ketones (13)** | 1-(4-Hydroxy-3-methoxyphenyl)-ethanone | C_9_H_10_O_3_(166.18) |  | Sweet, herbal | MS, GC, OD | SBV |  |
|  | 1-Octen-3-one | C_8_H_14_O(126.20) |  | Sweet | MS, GC, OD | ZAV |  |
|  | 1-Phenyl-1-propanone | C_9_H_10_O(134.18) |  | Pleasant honey scent | MS, GC, OD | SAV, ZAV |  |
|  | 2, 3-Butanedione | C_4_H_6_O_2_(86.09) |  | Buttery | MS, GC, OD | SAV, ZAV, BRV, KRV, JRV |  |
|  | 2, 5-Dimethyl-2,4-dihydroxy-3(2H)-furanone | C_6_H_8_O_4_(144.13) |  | Yoghurt | MS, GC, OD | ZAV |  |
|  | 2(5H)-Furanone | C_4_H_4_O_2_(84.07) |  | Burnt, wheat | MS, GC, OD | ZAV |  |
|  | 3-Acetoxy-2-butanone | C_6_H_10_O_3_(130.14) |  | Fruity | MS, GC, OD | ZAV, SAV, SBV, BRV |  |
|  | 2-Acetylpyrrole | C_6_H_7_NO(109.13) |  | Mold | MS, GC, OD | SBV, SAV |  |
|  | 2-Butanone | C_4_H_8_O(72.11) |  | Fruity, floral | MS, GC, OD | SAV, ZAV |  |
|  | Furfural acetone | C_8_H_8_O_2_(136.15) |  | - | MS, GC | ZAV |  |
|  | Acetoin | C_4_H_8_O_2_(88.11) |  | Butter, sweet, milky,creamy | MS, GC, OD | SAV, ZAV, SBV, KRV, BRV, JRV |  |
|  | Acetophenone | C_8_H_8_O( 120.15) |  | Floral,sweet,hawthorn,chery | MS, GC, OD | ZAV, SBV |  |
|  | Jasmone | C_11_H_16_O(164.25) |  | Geraniol, woody | MS, GC, OD | ZAV |  |
| **Lactones (4)** | γ-Butyrolactone | C_4_H_6_O_2_(86.09) |  | Cheese, fatty | MS, GC, OD | SBV |  |
|  | γ-Nonalactone | C_9_H_16_O_2_(156.23) |  | Sweet, coconut | MS, GC, OD | SAV, ZAV, SBV, BRV |  |
|  | Pantolactone | C_6_H_10_O_3_(130.14) |  | Floral | MS, GC, OD | ZAV |  |
|  | Sotolon | C_6_H_8_O_3_(128.13) |  | Caramel, sweet | MS, GC, OD | ZAV |  |
| **Phenols (8)** | 2-Methoxy-4-methylphenol | C_8_H_10_O_2_(138.17) |  | Spicy, Sweet ,clove, Woody, herbal | MS, GC, OD | SAV, ZAV , SBV, BRV |  |
|  | 2,4-Di-tert-butylphenol | C_14_H_22_O(206.33) |  | - | MS, GC | ZAV, BRV, JRV |  |
|  | 4-Ethylguaiacol | C_9_H_12_O_2_(152.19) |  | Smoky, spicy | MS, GC, OD | SAV, SBV, BRV |  |
|  | 4-Vinyl-gulaiacol | C_9_H_10_O_2_(150.18) |  | Woody, spicy, clove | MS, GC, OD | ZAV |  |
|  | 4-(1-Methylpropyl)- phenol | C_10_H_14_O(150.22) |  | - | MS, GC | SAV, ZAV |  |
|  | Guaiacol | C_7_H_8_O_2_(124.14) |  | Woody, smoky | MS, GC, OD | SAV, ZAV, SBV |  |
|  | Maltol | C_6_H_6_O_3_(126.11) |  | Caramel, sweet | MS, GC, OD | ZAV |  |
|  | Phenol | C_6_H_6_O(94.11) |  | Fruity, wine | MS, GC, OD | SAV, ZAV |  |
| **Pyrazines (6)** | 2,3-Dimethylpyrazine | C_6_H_8_N_2_(108.14) |  | Burt, roasted | MS, GC, OD | SAV, ZAV |  |
|  | 2,3,5-Trimethylpyrazine | C_7_H_10_N_2_(122.17) |  | Baked potato, earthy, nutty | MS, GC, OD | SAV, ZAV |  |
|  | 3-Ethyl-2,5-dimethylpyrazine | C_8_H_12_N_2_(136.20) |  | - | MS, GC,OD | SAV, ZAV |  |
|  | Ethyl pyrazine | C_6_H_8_N_2_(108.14) |  | Nutty | MS, GC, OD | SAV |  |
|  | Methylpyrazine | C_5_H_6_N_2_(94.12) |  | Roasted, nutty | MS, GC, OD | SAV |  |
|  | Tetramethylpyrazine | C_8_H_12_N_2_(136.20) |  | Smelly, nutty | MS, GC, OD | SAV, ZAV, SBV, BRV |  |
| **Furans (3)** | 2-Acetylfuran | C_6_H_6_O_2_(110.11) |  | - | MS, GC | SAV, ZAV |  |
|  | 2-Pentyl-furan | C_9_H_14_O(138.21) |  | Vegetable | MS, GC, OD | ZAV |  |
|  | 3-Phenyl-furan | C_10_H_8_O(144.17) |  | Earthy, green | MS, GC, OD | ZAV |  |

VOCs: Volatile organic compounds; MF:Molecular formula; MW: Molecular weight; ID: Identification; MS: Mass spectrometry; GC: gas chromatography; OD: Odor description at the olfactory detection port; E-nose: Electronic nose; SAV: Shanxi aged vinegar; ZAV: Zhenjiang aromatic vinegar; BRV: Beijing rice vinegar; KRV: Korean brown rice vinegar; JRV: Japanese rice vinegar; SBV: Sichuan bran vinegar; -: Not detected.

**Annex Table 3** **The main VOCs in fruit and cereal vinegars (210)**

| **Categories** | | | **VOCs** | **Fruit vinegars** | | | | | **Cereal vinegars** | | | | | |
| --- | --- | --- | --- | --- | --- | --- | --- | --- | --- | --- | --- | --- | --- | --- |
|  |  |  |  | **TBV** | **BV** | **SV** | **CV** | **PV** | **SAV** | **ZAV** | **SBV** | **BRV** | **KRV** | **JRV** |
| **I. Acids (27)** | Ⅰ-1 | | 2-Furoic acid | + | + | nd | nd | nd | nd | + | nd | nd | nd | nd |
|  | Ⅰ-2 | | 2-Methyl butanoic acid | nd | + | + | nd | nd | nd | nd | + | nd | nd | nd |
|  | Ⅰ-3 | | 2-Methyl propionic acid | + | + | + | nd | nd | nd | + | + | nd | + | + |
|  | Ⅰ-4 | | 3-Methyl butyric acid | + | + | + | nd | + | + | + | + | nd | + | + |
|  | Ⅰ-5 | | 3-(Methylthio)propionic acid | nd | nd | nd | nd | nd | nd | + | nd | nd | nd | nd |
|  | Ⅰ-6 | | 4-Methyl pentanoic acid | nd | nd | nd | nd | nd | nd | nd | + | nd | nd | nd |
|  | Ⅰ-7 | | 4-Oxopentanoic acid | + | + | nd | nd | nd | nd | nd | nd | nd | nd | nd |
|  | Ⅰ-8 | | 9-Hexadecenoic acid | + | nd | + | nd | nd | nd | nd | nd | nd | nd | nd |
|  | Ⅰ-9 | | 9-Octadecenoic acid | + | nd | nd | nd | nd | nd | nd | nd | nd | nd | nd |
|  | Ⅰ-10 | | Acetic acid | + | + | + | + | + | + | + | + | + | + | + |
|  | Ⅰ-11 | | Benzoic acid | + | + | + | nd | nd | nd | nd | nd | nd | nd | nd |
|  | Ⅰ-12 | | Butanoic acid | + | + | + | nd | nd | + | + | nd | nd | + | nd |
|  | Ⅰ-13 | | Decanoic acid | + | + | + | nd | nd | nd | nd | nd | nd | nd | nd |
|  | Ⅰ-14 | | (E*)*-But-2-enoic acid | + | + | nd | nd | nd | nd | nd | nd | nd | nd | nd |
|  | Ⅰ-15 | | Formic acid | + | nd | nd | nd | nd | nd | nd | nd | nd | nd | nd |
|  | Ⅰ-16 | | Hexanoic acid | + | + | + | nd | + | + | + | + | + | + | + |
|  | Ⅰ-17 | | Hexadecanoic acid | + | + | + | nd | nd | nd | nd | nd | nd | nd | nd |
|  | Ⅰ-18 | | Heptanoic acid | nd | + | + | nd | nd | nd | nd | + | nd | nd | nd |
|  | Ⅰ-19 | | Octanoic acid | + | + | + | nd | + | nd | + | + | + | + | + |
|  | Ⅰ-20 | | Octadecanoic acid | + | nd | nd | nd | nd | nd | nd | nd | nd | nd | nd |
|  | Ⅰ-21 | | Oleic acid | + | nd | nd | nd | nd | nd | nd | nd | nd | nd | nd |
|  | Ⅰ-22 | | Pentadecanoic acid | + | + | + | nd | nd | nd | nd | nd | nd | nd | nd |
|  | Ⅰ-23 | | Pentanoic acid | nd | + | + | nd | nd | + | + | nd | nd | nd | nd |
|  | Ⅰ-24 | | Phenylacetic acid | + | + | nd | nd | nd | nd | nd | nd | nd | nd | + |
|  | Ⅰ-25 | | Propanoic acid | + | + | + | nd | + | + | + | nd | nd | + | + |
|  | Ⅰ-26 | | Nonanoic acid | + | + | + | nd | nd | nd | nd | nd | nd | + | + |
|  | Ⅰ-27 | | Tetradecanoic acid | + | + | + | nd | nd | nd | nd | nd | nd | nd | nd |
| **Subtotal** | | | | **22** | **20** | **17** | **1** | **5** | **6** | **10** | **8** | **3** | **8** | **8** |
| **II. Alcohols (23)** | Ⅱ-1 | | 1-Hexanol | nd | nd | + | + | + | nd | + | nd | + | nd | nd |
|  | Ⅱ-2 | | 1-Pentanol | nd | nd | nd | nd | + | nd | nd | nd | nd | nd | nd |
|  | Ⅱ-3 | | 1-Propanol | nd | + | + | nd | nd | + | nd | nd | nd | nd | nd |
|  | Ⅱ-4 | | 2, 3-Butanediol | + | + | + | nd | + | + | + | nd | nd | nd | nd |
|  | Ⅱ-5 | | 2-Butanol | nd | nd | nd | nd | nd | + | + | nd | nd | nd | nd |
|  | Ⅱ-6 | | 2-Butoxy-ethanol | nd | nd | nd | nd | + | nd | nd | nd | nd | nd | nd |
|  | Ⅱ-7 | | 2-Ethyl-1-hexanol | nd | nd | nd | nd | + | + | + | + | nd | nd | nd |
|  | Ⅱ-8 | | 2-Ethyl-2-hexanol | nd | nd | nd | nd | nd | nd | + | nd | nd | nd | nd |
|  | Ⅱ-9 | | 2-Furanmethanol | nd | + | + | nd | nd | + | + | + | nd | nd | nd |
|  | Ⅱ-10 | | 2-Hexanol | + | + | nd | nd | nd | nd | nd | nd | nd | nd | nd |
|  | Ⅱ-11 | | 2-Methyl-1-propanol | + | + | + | nd | + | + | + | nd | + | + | + |
|  | Ⅱ-12 | | 2-Methyl-1-butanol | + | + | + | + | nd | nd | nd | nd | nd | + | + |
|  | Ⅱ-13 | | 3-Ethoxy-1-propanol | + | nd | nd | nd | nd | nd | nd | nd | nd | nd | nd |
|  | Ⅱ-14 | | 3-Methyl-1-butanol | + | + | + | + | + | + | + | nd | + | + | + |
|  | Ⅱ-15 | | 3-Methylthio-propanol | nd | + | + | nd | + | + | + | + | nd | nd | nd |
|  | Ⅱ-16 | | Benzyl alcohol | + | + | + | nd | nd | + | + | + | nd | nd | nd |
|  | Ⅱ-17 | | Borneol | + | + | + | nd | nd | nd | nd | nd | nd | nd | nd |
|  | Ⅱ-18 | | *cis*-3-hexen-1-ol | nd | + | + | nd | nd | nd | nd | nd | nd | nd | nd |
|  | Ⅱ-19 | | Ethanol | nd | + | + | nd | nd | + | + | nd | + | + | + |
|  | Ⅱ-20 | | Fenchyl alcohol | + | + | + | nd | nd | nd | nd | nd | nd | nd | nd |
|  | Ⅱ-21 | | Methanol | nd | + | + | nd | nd | nd | nd | nd | nd | nd | nd |
|  | Ⅱ-22 | | Octanol | nd | nd | nd | nd | + | nd | nd | nd | nd | nd | nd |
|  | Ⅱ-23 | | Phenylethyl alcohol | + | + | + | nd | + | + | + | + | + | + | + |
| **Subtotal** | | | | **10** | **15** | **15** | **3** | **10** | **11** | **12** | **5** | **5** | **5** | **5** |
| **III. Aldehydes (32)** | Ⅲ-1 | | 1H-pyrrole-2-carboxaldehyde | nd | nd | nd | nd | nd | nd | nd | + | nd | nd | nd |
|  | Ⅲ-2 | | 2-Hexenal | nd | nd | nd | + | nd | nd | nd | nd | nd | nd | nd |
|  | Ⅲ-3 | | 2-Methyl butanal | nd | nd | nd | nd | nd | + | + | nd | nd | nd | nd |
|  | Ⅲ-4 | | 2-Methyl propanal | nd | nd | nd | nd | nd | + | + | nd | nd | nd | nd |
|  | Ⅲ-5 | | 2-Methyl benzaldehyde | nd | nd | nd | nd | nd | nd | nd | nd | nd | + | + |
|  | Ⅲ-6 | | 2-Phenyl-2-butenal | nd | nd | nd | nd | nd | nd | + | + | nd | nd | nd |
|  | Ⅲ-7 | | 2-Phenyl-3-(2-furyl)-propenal | nd | nd | nd | nd | nd | nd | nd | + | nd | nd | nd |
|  | Ⅲ-8 | | 2-Phenylacetaldehyde | nd | + | nd | nd | nd | + | + | + | nd | nd | nd |
|  | Ⅲ-9 | | 3-Methyl benzaldehyde | nd | nd | nd | nd | + | nd | nd | nd | nd | nd | nd |
|  | Ⅲ-10 | | 3-Methyl butanal | nd | + | + | nd | nd | + | + | + | nd | + | nd |
|  | Ⅲ-11 | | 4-Methyl benzaldehyde | nd | nd | nd | nd | nd | nd | nd | nd | nd | + | + |
|  | Ⅲ-12 | | 4-Methoxybenzaldehyde | nd | nd | nd | nd | nd | + | + | nd | nd | nd | nd |
|  | Ⅲ-13 | | 5-Acetoxymethyl-2-furaldehyde | + | + | + | nd | nd | nd | nd | nd | nd | nd | nd |
|  | Ⅲ-14 | | 5-Ethoxymethylfurfural | + | + | + | nd | nd | nd | nd | nd | nd | nd | nd |
|  | Ⅲ-15 | | 5-Hydroxymethylfurfural | + | + | + | nd | nd | nd | + | + | nd | nd | nd |
|  | Ⅲ-16 | | 5-Methyl-2-phenyl-2-hexenal | nd | nd | nd | nd | nd | nd | nd | + | nd | nd | nd |
|  | Ⅲ-17 | | 5-Methyl furfural | + | + | + | nd | nd | + | + | + | nd | nd | nd |
|  | Ⅲ-18 | | Acetaldehyde | nd | + | + | nd | nd | + | + | nd | nd | + | + |
|  | Ⅲ-19 | | Benzaldehyde | + | + | + | + | nd | + | + | nd | nd | + | + |
|  | Ⅲ-20 | | Cinnamaldehyde | nd | nd | nd | nd | nd | + | + | nd | nd | nd | nd |
|  | Ⅲ-21 | | Decanal | nd | + | nd | nd | nd | nd | + | nd | nd | nd | nd |
|  | Ⅲ-22 | | Dodecanal | nd | nd | nd | nd | nd | nd | nd | nd | nd | + | + |
|  | Ⅲ-23 | | (E)-3-(furan-2-yl)-2-methylprop-2-enal | nd | nd | nd | nd | nd | nd | nd | + | nd | nd | nd |
|  | Ⅲ-24 | | Furfural | + | + | + | nd | nd | + | + | + | + | + | + |
|  | Ⅲ-25 | | Hexanal | nd | + | + | + | nd | + | + | nd | nd | nd | nd |
|  | Ⅲ-26 | | Methional | nd | nd | + | nd | nd | + | + | nd | nd | nd | nd |
|  | Ⅲ-27 | | Nonanal | nd | + | + | nd | nd | nd | + | nd | nd | nd | nd |
|  | Ⅲ-28 | | Octanal | + | + | + | nd | nd | nd | + | nd | nd | nd | nd |
|  | Ⅲ-29 | | Propionaldehyde | nd | nd | nd | nd | nd | nd | nd | nd | nd | + | + |
|  | Ⅲ-30 | | Safranal | nd | nd | nd | nd | nd | nd | + | nd | nd | nd | nd |
|  | Ⅲ-31 | | Vanillin | + | + | + | nd | nd | + | + | + | nd | nd | nd |
|  | Ⅲ-32 | | (Z)-2-nonenal | nd | nd | nd | nd | nd | + | nd | nd | nd | nd | nd |
| **Subtotal** | | | | **8** | **14** | **13** | **3** | **1** | **14** | **19** | **11** | **1** | **8** | **7** |
| **IV. Esters (64)** | Ⅳ-1 | | 1, 2-Propanediol diacetate | + | + | + | nd | nd | nd | nd | nd | nd | nd | nd |
|  | Ⅳ-2 | | 1, 3-Propanediol diacetate | nd | + | nd | nd | nd | nd | nd | nd | nd | nd | nd |
|  | Ⅳ-3 | | 2-Butyl acetate | nd | nd | nd | nd | nd | + | + | nd | nd | nd | nd |
|  | Ⅳ-4 | | 2-Furanmethanol acetate | nd | nd | nd | nd | nd | nd | + | + | nd | nd | nd |
|  | Ⅳ-5 | | 2-Methylpropyl acetate | + | + | + | + | + | + | + | nd | + | + | + |
|  | Ⅳ-6 | | 2-Phenylethyl acetate | + | + | + | nd | + | + | + | + | + | + | + |
|  | Ⅳ-7 | | 2-Methylbutyl acetate | nd | + | nd | + | nd | nd | nd | nd | nd | nd | nd |
|  | Ⅳ-8 | | 3-(Acetyloxy) butan-2-yl acetate | + | + | + | nd | nd | nd | + | nd | nd | nd | nd |
|  | Ⅳ-9 | | 3-Methylbutyl acetate | + | + | + | + | + | + | + | nd | + | + | + |
|  | Ⅳ-10 | | 3-Methylbutyl pentanoate | nd | nd | nd | + | nd | nd | nd | nd | nd | nd | nd |
|  | Ⅳ-11 | | 3-(Methylthio)propyl acetate | nd | nd | nd | nd | nd | + | + | nd | nd | nd | nd |
|  | Ⅳ-12 | | 4-Methyl-2-pentyl acetate | + | + | + | nd | nd | nd | nd | nd | nd | nd | nd |
|  | Ⅳ-13 | | Amyl acetate | nd | nd | + | nd | nd | nd | nd | nd | nd | nd | nd |
|  | Ⅳ-14 | | Benzyl acetate | + | + | + | nd | nd | nd | nd | nd | nd | nd | nd |
|  | Ⅳ-15 | | Bornyl acetate | + | nd | + | nd | nd | nd | nd | nd | nd | nd | nd |
|  | Ⅳ-16 | | Butyl acetate | nd | + | + | nd | nd | nd | nd | nd | nd | nd | nd |
|  | Ⅳ-17 | | Butyl benzoate | nd | nd | nd | nd | nd | nd | + | nd | nd | nd | nd |
|  | Ⅳ-18 | | Diethyl succinate | + | + | + | nd | nd | + | + | nd | + | + | + |
|  | Ⅳ-19 | | Dihydroxymethyl jasmonate | + | + | + | nd | nd | nd | nd | nd | nd | nd | nd |
|  | Ⅳ-20 | | Ethyl 2-hydroxy-4-methyl pentanoate | nd | nd | nd | nd | nd | nd | + | nd | nd | nd | nd |
|  | Ⅳ-21 | | Ethyl 2-methylbutyrate | nd | nd | + | + | nd | nd | nd | nd | nd | nd | + |
|  | Ⅳ-22 | | Ethyl-2-methylpropanoate | nd | + | + | + | nd | nd | nd | nd | nd | nd | nd |
|  | Ⅳ-23 | | Ethyl 3-hydroxyhexanoate | nd | nd | nd | nd | + | nd | nd | nd | nd | nd | nd |
|  | Ⅳ-24 | | Ethyl 3-hydroxybutanoate | nd | nd | + | nd | nd | nd | nd | nd | nd | nd | nd |
|  | Ⅳ-25 | | Ethyl 3-methylbutyrate | + | + | + | + | nd | nd | + | nd | nd | nd | nd |
|  | Ⅳ-26 | | Ethyl 3-methylthio-propanoate | nd | nd | nd | nd | + | nd | nd | nd | nd | nd | nd |
|  | Ⅳ-27 | | Ethyl 4-acetoxybutanoate | nd | nd | nd | nd | nd | nd | + | nd | nd | nd | nd |
|  | Ⅳ-28 | | Ethyl acetate | + | + | + | + | + | + | + | nd | + | + | + |
|  | Ⅳ-29 | | Ethyl acetoacetate | nd | + | nd | nd | nd | nd | nd | nd | nd | nd | nd |
|  | Ⅳ-30 | | Ethyl benzoate | nd | nd | + | nd | + | + | + | nd | + | nd | nd |
|  | Ⅳ-31 | | Ethyl butanoate | + | + | + | nd | + | nd | nd | nd | nd | nd | nd |
|  | Ⅳ-32 | | Ethyl decanoate | + | + | + | nd | + | nd | + | nd | nd | nd | nd |
|  | Ⅳ-33 | | Ethyl furoate | nd | + | + | nd | nd | nd | nd | nd | nd | nd | nd |
|  | Ⅳ-34 | | Ethyl heptanoate | nd | nd | + | nd | + | nd | nd | nd | nd | nd | nd |
|  | Ⅳ-35 | | Ethyl hexanoate | + | + | + | + | + | nd | + | nd | + | nd | nd |
|  | Ⅳ-36 | | Ethyl valerate | nd | + | + | nd | nd | nd | nd | nd | nd | nd | nd |
|  | Ⅳ-37 | | Ethyl lactate | + | + | + | nd | nd | + | + | + | nd | + | nd |
|  | Ⅳ-38 | | Ethyl levulinate | + | + | + | nd | nd | nd | nd | nd | nd | nd | nd |
|  | Ⅳ-39 | | Ethyl nonanoate | nd | nd | + | nd | nd | nd | nd | nd | nd | nd | nd |
|  | Ⅳ-40 | | Ethyl octanoate | + | + | + | nd | + | + | + | nd | nd | nd | nd |
|  | Ⅳ-41 | | Ethyl phenylacetate | + | + | + | nd | + | + | + | nd | + | + | nd |
|  | Ⅳ-42 | | Ethyl propanoate | nd | + | + | + | nd | + | nd | nd | nd | + | + |
|  | Ⅳ-43 | | Ethyl salicylate | nd | nd | + | nd | nd | nd | nd | nd | nd | nd | nd |
|  | Ⅳ-44 | | Ethyl vanillate | + | + | + | nd | nd | nd | nd | nd | nd | nd | nd |
|  | Ⅳ-45 | | Geranyl acetate | + | + | + | nd | nd | nd | nd | nd | nd | nd | nd |
|  | Ⅳ-46 | | Hexyl acetate | + | + | + | + | nd | nd | + | nd | nd | nd | nd |
|  | Ⅳ-47 | | Isobutyl isothiocyanate | + | + | + | nd | nd | nd | nd | nd | nd | nd | nd |
|  | Ⅳ-48 | | Methyl 2-methylbutyrate | nd | nd | nd | nd | nd | nd | + | nd | nd | nd | nd |
|  | Ⅳ-49 | | [Methyl 2-methylpropanoate](https://www.chembk.com/en/chem/methyl%202-methylpropanoate) | nd | nd | nd | nd | nd | nd | nd | nd | nd | + | nd |
|  | Ⅳ-50 | | Methyl 3-hydroxy-2-methyl-butanoate | nd | nd | nd | nd | + | nd | nd | nd | nd | nd | nd |
|  | Ⅳ-51 | | Methyl 3-methylthio-propanoate | nd | nd | nd | nd | + | nd | nd | nd | nd | nd | nd |
|  | Ⅳ-52 | | Methyl acetate | nd | + | + | nd | nd | + | + | nd | nd | + | + |
|  | Ⅳ-53 | | Methyl butyrate | nd | nd | nd | nd | nd | nd | + | nd | nd | nd | nd |
|  | Ⅳ-54 | | Methyl cinnamate | nd | nd | nd | nd | nd | + | + | nd | nd | nd | nd |
|  | Ⅳ-55 | | Methyl furan-2-carboxylate | nd | + | nd | nd | nd | nd | + | nd | nd | nd | nd |
|  | Ⅳ-56 | | Methyl hexanoate | + | nd | + | nd | nd | nd | nd | nd | nd | nd | nd |
|  | Ⅳ-57 | | Methyl salicylate | + | + | + | nd | nd | nd | nd | nd | nd | nd | nd |
|  | Ⅳ-58 | | Monomethyl succinate | nd | nd | nd | nd | nd | nd | + | nd | nd | nd | nd |
|  | Ⅳ-59 | | Neryl acetate | + | + | + | nd | nd | nd | nd | nd | nd | nd | nd |
|  | Ⅳ-60 | | N-pentyl acetate | nd | nd | nd | nd | nd | nd | + | nd | nd | nd | nd |
|  | Ⅳ-61 | | Propyl acetate | nd | + | + | nd | nd | + | + | nd | nd | nd | nd |
|  | Ⅳ-62 | | Vinyl acetate | nd | nd | nd | + | nd | nd | nd | nd | nd | + | + |
|  | Ⅳ-63 | | (E)-2-Hexen-1-ol acetate | + | nd | + | nd | nd | nd | nd | nd | nd | nd | nd |
|  | Ⅳ-64 | | (Z)-3-Hexen-1-ol acetate | + | + | + | nd | nd | nd | nd | nd | nd | nd | nd |
| **Subtotal** | | | | **28** | **36** | **42** | **12** | **15** | **15** | **28** | **3** | **8** | **11** | **9** |
| **V. Ketones (30)** | Ⅴ-1 | | 1-(2,3,6-Trimethylphenyl)-3-buten-2-one | + | + | + | nd | nd | nd | nd | nd | nd | nd | nd |
|  | Ⅴ-2 | | 1-(4-Hydroxy-3-methoxyphenyl)-ethanone | nd | nd | nd | nd | nd | nd | nd | + | nd | nd | nd |
|  | Ⅴ-3 | | 1-(5-Methyl-2-furyl)-1-propanone | + | + | + | nd | nd | nd | nd | nd | nd | nd | nd |
|  | Ⅴ-4 | | 1-Octen-3-one | nd | nd | nd | nd | nd | nd | + | nd | nd | nd | nd |
|  | Ⅴ-5 | | 1-Phenyl-1-propanone | nd | nd | nd | nd | nd | + | + | nd | nd | nd | nd |
|  | Ⅴ-6 | | 2, 3-Butanedione | + | + | + | nd | nd | + | + | nd | + | + | + |
|  | Ⅴ-7 | | 2, 5-Dimethyl-2,4-dihydroxy-3(2H)-furanone | nd | nd | nd | nd | nd | nd | + | nd | nd | nd | nd |
|  | Ⅴ-8 | | 2-Acetylpyrrole | nd | nd | nd | nd | nd | + | nd | + | nd | nd | nd |
|  | Ⅴ-9 | | 2-Butanone | nd | nd | nd | nd | nd | + | + | nd | nd | nd | nd |
|  | Ⅴ-10 | | 2-Hydroxy-3-methyl-cyclopent-2-en-1-one | + | + | nd | nd | nd | nd | nd | nd | nd | nd | nd |
|  | Ⅴ-11 | | 2-Methyloxolan-3-one | + | + | nd | nd | nd | nd | nd | nd | nd | nd | nd |
|  | Ⅴ-12 | | 2-Methyl-3-hydroxy-4-pyrone | nd | nd | + | nd | nd | nd | nd | nd | nd | nd | nd |
|  | Ⅴ-13 | | 2-Nonanone | nd | nd | nd | nd | + | nd | nd | nd | nd | nd | nd |
|  | Ⅴ-14 | | 2(5H)-Furanone | nd | nd | nd | nd | nd | nd | + | nd | nd | nd | nd |
|  | Ⅴ-15 | | 3,5-Dihydroxy-2-methyl-(4H)-pyran-4-one | + | + | nd | nd | nd | nd | nd | nd | nd | nd | nd |
|  | Ⅴ-16 | | 3-Acetoxy-2-butanone | nd | nd | nd | nd | nd | + | + | + | + | nd | nd |
|  | Ⅴ-17 | | 3-Hydroxy-2-butanone | + | + | + | nd | + | + | + | + | + | + | + |
|  | Ⅴ-18 | | 3-Hydroxy-2-pentanone | nd | nd | + | nd | nd | nd | nd | nd | nd | nd | nd |
|  | Ⅴ-19 | | 4-Methyl heptanone | nd | nd | nd | nd | + | nd | nd | nd | nd | nd | nd |
|  | Ⅴ-20 | | 5,6-Dihydro-4-methyl-(2H)-pyran-2-one | + | + | nd | nd | nd | nd | nd | nd | nd | nd | nd |
|  | Ⅴ-21 | | 5-Methyl-3-hexanone | + | + | + | nd | nd | nd | nd | nd | nd | nd | nd |
|  | Ⅴ-22 | | 6-Methyl 5-hepten-2-one | nd | + | nd | + | nd | nd | nd | nd | nd | nd | nd |
|  | Ⅴ-23 | | Acetophenone | nd | nd | + | nd | nd | nd | + | + | nd | nd | nd |
|  | Ⅴ-24 | | Benzophenone | + | + | + | nd | nd | nd | nd | nd | nd | nd | nd |
|  | Ⅴ-25 | | Camphor | nd | nd | nd | + | nd | nd | nd | nd | nd | nd | nd |
|  | Ⅴ-26 | | Furfural acetone | nd | nd | nd | nd | nd | nd | + | nd | nd | nd | nd |
|  | Ⅴ-27 | | Isovalerone | + | + | + | nd | nd | nd | nd | nd | nd | nd | nd |
|  | Ⅴ-28 | | Jasmone | nd | nd | nd | nd | nd | nd | + | nd | nd | nd | nd |
|  | Ⅴ-29 | | Neryl acetone | nd | + | nd | nd | nd | nd | nd | nd | nd | nd | nd |
|  | Ⅴ-30 | | β-Damascenone | nd | nd | + | nd | nd | nd | nd | nd | nd | nd | nd |
| **Subtotal** | | | | **11** | **13** | **11** | **2** | **3** | **6** | **11** | **5** | **3** | **2** | **2** |
| **VI. Lactones (12)** | Ⅵ-1 | | α-Methyl-γ-crotonolactone | + | + | + | nd | nd | nd | nd | nd | nd | nd | nd |
|  | Ⅵ-2 | | *Trans*-β-methyl-γ-octalactone | nd | + | + | nd | nd | nd | nd | nd | nd | nd | nd |
|  | Ⅵ-3 | | *cis*-β-methyl-γ-octalactone | nd | + | + | nd | nd | nd | nd | nd | nd | nd | nd |
|  | Ⅵ-4 | | γ-Butyrolactone | + | + | + | nd | nd | nd | nd | + | nd | nd | nd |
|  | Ⅵ-5 | | γ-Decalactone | nd | nd | + | nd | nd | nd | nd | nd | nd | nd | nd |
|  | Ⅵ-6 | | γ-Heptalactone | + | + | + | nd | nd | nd | nd | nd | nd | nd | nd |
|  | Ⅵ-7 | | γ-Nonalactone | nd | nd | nd | nd | nd | + | + | + | + | nd | nd |
|  | Ⅵ-8 | | δ-2-Decenolactone | + | + | + | nd | nd | nd | nd | nd | nd | nd | nd |
|  | Ⅵ-9 | | δ-Laurolactone | + | + | + | nd | nd | nd | nd | nd | nd | nd | nd |
|  | Ⅵ-10 | | δ-Decalactone | + | + | + | nd | nd | nd | nd | nd | nd | nd | nd |
|  | Ⅵ-11 | | Pantolactone | nd | nd | nd | nd | nd | nd | + | nd | nd | nd | nd |
|  | Ⅵ-12 | | Sotolon | nd | nd | + | nd | nd | nd | + | nd | nd | nd | nd |
| **Subtotal** | | | | **6** | **8** | **10** | **0** | **0** | **1** | **3** | **2** | **1** | **0** | **0** |
| **VII. Phenols (12)** | Ⅶ-1 | | 2, 4-Ditertbutyl phenol | + | + | + | nd | nd | nd | nd | nd | + | nd | + |
|  | Ⅶ-2 | | 2-Methoxy-4-methylphenol | nd | nd | nd | nd | nd | + | + | + | + | nd | nd |
|  | Ⅶ-3 | | 4-(1-Methylpropyl)- phenol | nd | nd | nd | nd | nd | + | + | nd | nd | nd | nd |
|  | Ⅶ-4 | | 4-Acetyl-2-methylphenol | + | + | + | nd | nd | nd | nd | nd | nd | nd | nd |
|  | Ⅶ-5 | | 4-Ethyl phenol | + | + | + | nd | + | nd | nd | nd | nd | nd | nd |
|  | Ⅶ-6 | | 4-Ethyl guaiacol | + | + | + | nd | nd | + | nd | + | + | nd | nd |
|  | Ⅶ-7 | | 4-Vinyl guaiacol | nd | nd | + | nd | + | nd | + | nd | nd | nd | nd |
|  | Ⅶ-8 | | 4-Vinyl phenol | nd | nd | nd | nd | + | nd | nd | nd | nd | nd | nd |
|  | Ⅶ-9 | | Eugenol | + | + | + | nd | + | nd | nd | nd | nd | nd | nd |
|  | Ⅶ-10 | | Guaiacol | nd | + | + | nd | nd | + | + | + | nd | nd | nd |
|  | Ⅶ-11 | | Maltol | + | + | nd | nd | nd | nd | + | nd | nd | nd | nd |
|  | Ⅶ-12 | | Phenol | + | + | + | nd | + | + | + | nd | nd | nd | nd |
| **Subtotal** | | | | **7** | **8** | **8** | **0** | **5** | **5** | **6** | **3** | **3** | **0** | **1** |
| **VIII. Pyrazines (7)** | Ⅷ-1 | | 2, 3-Dimethylpyrazine | nd | nd | nd | nd | nd | + | + | nd | nd | nd | nd |
|  | Ⅷ-2 | | 2, 5-Dimethylpyrazine | nd | nd | nd | nd | + | nd | nd | nd | nd | nd | nd |
|  | Ⅷ-3 | | 2, 3, 5-Trimethylpyrazine | nd | nd | nd | nd | nd | + | + | nd | nd | nd | nd |
|  | Ⅷ-4 | | 3-Ethyl-2, 5-dimethylpyrazine | nd | nd | nd | nd | nd | + | + | nd | nd | nd | nd |
|  | Ⅷ-5 | | Ethylpyrazine | nd | nd | nd | nd | nd | + | nd | nd | nd | nd | nd |
|  | Ⅷ-6 | | Methylpyrazine | nd | nd | nd | nd | nd | + | nd | nd | nd | nd | nd |
|  | Ⅷ-7 | | Tetramethyl pyrazine | nd | nd | nd | nd | nd | + | + | + | + | nd | nd |
| **Subtotal** | | | | **0** | **0** | **0** | **0** | **1** | **6** | **4** | **1** | **1** | **0** | **0** |
| **IX. Furans (3)** | Ⅸ-1 | 2-Acetylfuran | | + | + | + | nd | nd | + | + | nd | nd | nd | nd |
|  | Ⅸ-2 | 2-Pentyl-furan | | nd | nd | nd | nd | nd | nd | + | nd | nd | nd | nd |
|  | Ⅸ-3 | 3-Phenyl-furan | | nd | nd | nd | nd | nd | nd | + | nd | nd | nd | nd |
| **Subtotal** | | | | **1** | **1** | **1** | **0** | **0** | **1** | **3** | **0** | **0** | **0** | **0** |
| **Total** | | | | **93** | **115** | **117** | **21** | **40** | **65** | **96** | **38** | **25** | **34** | **32** |

TBV: Traditional balsamic vinegar; BV: Balsamic vinegar; SV: Sherry vinegar; CV: Cider vinegar; PV: Pineapple vinegar; SAV: Shanxi aged vinegar; ZAV: Zhenjiang aromatic vinegar; BRV: Beijing rice vinegar;

KRV: Korean brown rice vinegar; JRV: Japanese rice vinegar; SBV: Sichuan bran vinegar; nd: Not detected.

**References**

Acena, L., Vera, L., Guasch, J., Busto, O. and Mestres, M. (2011). Chemical characterization of commercial Sherry vinegar aroma by headspace solid-phase microextraction and gas chromatography-olfactometry. *J. Agric. Food Chem.* 59(8), 4062–4070. doi: 10.1021 /jf104763u

Al-Dalai, S.A., Zheng, F., Sun, B. and Chen, F. (2019a). Comparison of aroma profiles of traditional and modern Zhenjiang aromatic vinegars and their changes during the vinegar aging by SPME-GC-MS and GC-O. *Food Anal Methods.* 12(2), 544-557. doi: 10.1007/s12161-018-1385-9

Al-Dalai, S.A., Zheng, F., Sun, B. and Chen, F. (2020). Characterization and comparison of aroma profiles and aroma-active compounds between traditional and modern Sichuan vinegars by molecular sensory science. *J. Agric. Food Chem.* 68(18), 1-44. doi: 10.1021/acs. jafc.0c00470

Al-Dalali, S., Zheng, F., Li, H., Huang, M. and Chen, F. (2019b). Characterization of volatile compounds in three commercial Chinese vinegars by SPME-GC-MS and GC-O. *LWT*. 112, 1-11. doi: 10.1016/j.lwt.2019.108264

Callejón, R.M., Morales, M., Troncoso, A.M., and Silva Ferreira, A.C. (2008). Targeting key aromatic substances on the typical aroma of Sherry vinegar. *J. Agric. Food Chem.* 56, 6631-6639. doi: 10.1021/ jf703 636e

Chung, N., Jo, Y., Joe, M., Jeong, M., Jeong, Y., and Kwon, J. (2017). Rice vinegars of different origins: discriminative characteristics based on solid-phase microextraction and gas chromatography with mass spectrometry, an electronic nose, electronic tongue and sensory evaluation. *J. I. Brewing.* 123, 159–166. doi: 10.1002/jib.406

Cirlini, M., Caligiani, A., Palla, L., and Palla, G. (2011). HS-SPME/GC–MS and chemometrics for the classification of Balsamic vinegars of Modena of different maturation and ageing. *Food Chem*. 124(4), 1678–1683. doi: 10.10 16/j.foodchem.2010.07.065

Corsini, L., Castro, R., Barroso, C., and Durán-Guerrero, E. (2019). Characterization by gas chromatography-olfactometry of the most odouractive compounds in Italian balsamic vinegars with geographical indication. *Food Chem*. 272, 702–708. doi: 10.1016/j.foodchem.2018.08.100

Giordano, L., Calabrese, R., Davoli, R., and Rotilio, D. (2003). Quantitative analysis of 2-furfural and 5-methylfurfural in different Italian vinegars by headspace solid-phase microextraction coupled to gas chromatography–mass spectrometry using isotope dilution. *J. Chromatogr. A*. 1017(1-2), 141–149. doi: 10.1016/j.chroma.2003.08.029

Liang, J., Xie, J., Hou, L., Zhao, M., Zhao, J., Cheng, J., et al. (2016). Aroma constituents in Shanxi aged vinegar before and after aging. *J. Agric. Food Chem.* 64(40), 7597−7605. doi: 10.1021/acs.jafc.6b03019

Marrufo-Curtido, A., Cejudo-Bastante, M.J., Durán-Guerrero, E., Castro-Mejías, R., Natera-Marín, R., Chinnici, F., et al. (2012). Characterization and differentiation of high-quality vinegars by stir bar sorptive extraction coupled to gas chromatography-mass spectrometry (SBSE-GC-MS). *LWT.* 47(2), 332-341. [doi: 10.1016/j.lwt.2012.01.028](https://doi.org/10.1016/j.lwt.2012.01.028)

Pinu, F.R., De Carvalho-Silva, S., Trovatti Uetanabaro, A.P. and Villas-Boas, S.G. (2016). Vinegar metabolomics: An explorative study of commercial balsamic vinegars using gas chromatography-mass spectrometry. *Metab*. 6(3), 1-15. doi: 10.3390/metabo6030022

Roda, A., Lucini, L., Torchio, F., Dordoni, R., De Faveri, D.M. and Lambri, M. (2017). Metabolite profiling and volatiles of pineapple wine and vinegar obtained from pineapple waste. *Food Chem.* 229(15), 734–742. doi: 10.1016/j.foodchem.2017.02.111

Truta, D. M., Tofana, M., Socaci, S.A. and Mudura, E., (2010). Comparison of different GC-MS methods used for the volatile compounds identification from commercial apple vinegar. *447 Bulletin UASVM Agriculture*. 67(2), 447-451. doi: 10.15835/buasvmcn-agr:5198

Wang, Z., Li, T., Liu, F., Zhang, C., Ma, H., Wang, L., et al. (2017). Eﬀects of ultrasonic treatment on the maturation of Zhenjiang vinegar. *Ultrason Sonochem*. 39, 272–280. doi: 10.1016 /j.ultsonch.2017.04.020

Xiao, Z., Dai, S., Niu, Y., Yu, H., Zhu, J., Tian, H., et al. (2011). Discrimination of Chinese vinegars based on head space solid-phase micro extraction-gas chromatography mass spectrometry of volatile compounds and multivariate analysis. *J. Food Sci.* 76(8), 1125-1135. doi: 10.1111/j.1750-3841.2011.02356.x

Zhang, X., Wang, P., Xu, D., Wang, W., and Zhao, Y. (2019). Aroma patterns of Beijing rice vinegar and their potential biomarker for traditional Chinese cereal vinegars. *Food Res. Int.* 119, 398–410. doi: 10.1016/j. foodres.2019.02.008

Zhao, C., Xia, T., Du, P., Duan, W., Zhang, B., and Zhang, J. (2018). Chemical composition and antioxidant characteristic of traditional and industrial Zhenjiang aromatic vinegars during the aging process. *Molecules.* 23(11), 1-17. doi: 10.3390/molecules23112949

Zhou, Z., Jian, D., Gong, M., Zhu, S., Li, G., Zhang, S., et al. (2020). Characterization of the key aroma compounds in aged Zhenjiang aromatic vinegar by gas chromato-graphy-olfactometry-mass spectrometry, quantitative measurements, aroma recombination and omission experiments. *Food Res. Int.* 136, 109434. doi: 10.1016/j.foodres.2020. 109434

Zhou, Z., Liu, S., Kong, X., Ji, Z., Han, X., Wu, J., et al. (2017). Elucidation of the aroma compositions of Zhenjiang aromatic vinegar using comprehendsive two dimensional gas chromatography coupled totime-of-flight massspectrometry and gas chromatography-olfactometry. *J. Chromatogr. A.* 1487, 218–226. doi: 10.1016/j.chroma.2017.01.014

Zhu, H., Zhu, J., Wang, L., and Li, Z. (2016). Development of a SPME-GC-MS method for the determination of volatile compounds in Shanxi aged vinegar and its analytical characterization by aroma wheel. *J. Food Sci. Technol.* 53(1), 171–183. [doi: 10.1007/s13197-015-2035-5](https://doi.org/10.1007/s13197-015-2035-5)

Zhu, Y., Zhang, F., Zhang, C., Yang, L., Fan, G., Xu, Y., et al. (2018). Dynamic microbial succession of Shanxi aged vinegar and its correlation with favor metabolites during different stages of acetic acid fermentation. *Sci. Rep.* 8(1), 1-10. doi: 10.1038/s41598-018-26787-6
